# Involvement of Novel Adipokines, Chemerin, Visfatin, Resistin and Apelin in Reproductive Functions in Normal and Pathological Conditions in Humans and Animal Models

**DOI:** 10.3390/ijms20184431

**Published:** 2019-09-09

**Authors:** Anthony Estienne, Alice Bongrani, Maxime Reverchon, Christelle Ramé, Pierre-Henri Ducluzeau, Pascal Froment, Joëlle Dupont

**Affiliations:** 1 INRA UMR 85 Physiologie de la Reproduction et des Comportements, F-37380 Nouzilly, France; 2 CNRS UMR 7247 Physiologie de la Reproduction et des Comportements, F-37380 Nouzilly, France; 3 Université François Rabelais de Tours F-37041 Tours, France; 4 IFCE, F-37380 Nouzilly, France; 5 SYSAAF-Syndicat des Sélectionneurs Avicoles et Aquacoles Français, Centre INRA Val de Loire, F-37380 Nouzilly, France; 6 Internal Medicine Department, Unit of Endocrinology, CHRU Tours, F-37044 Tours, France

**Keywords:** ovary, testis, adipose tissue, polycystic ovary syndrome, preeclempsia, gestational diabetes, testicular pathologies

## Abstract

It is well known that adipokines are endocrine factors that are mainly secreted by white adipose tissue. Their central role in energy metabolism is currently accepted. More recently, their involvement in fertility regulation and the development of some reproductive disorders has been suggested. Data concerning the role of leptin and adiponectin, the two most studied adipokines, in the control of the reproductive axis are consistent. In recent years, interest has grown about some novel adipokines, chemerin, visfatin, resistin and apelin, which have been found to be strongly associated with obesity and insulin-resistance. Here, we will review their expression and role in male and female reproduction in humans and animal models. According to accumulating evidence, they could regulate the secretion of GnRH (Gonadotropin-Releasing Hormone), gonadotropins and steroids. Furthermore, their expression and that of their receptors (if known), has been demonstrated in the human and animal hypothalamo-pituitary-gonadal axis. Like leptin and adiponectin, these novel adipokines could thus represent metabolic sensors that are able to regulate reproductive functions according to energy balance changes. Therefore, after investigating their role in normal fertility, we will also discuss their possible involvement in some reproductive troubles known to be associated with features of metabolic syndrome, such as polycystic ovary syndrome, gestational diabetes mellitus, preeclampsia and intra-uterine growth retardation in women, and sperm abnormalities and testicular pathologies in men.

## 1. Introduction

Nowadays, it is well known that there is a close link between metabolism and reproductive function [1,2]. Adipose tissue is now considered as an endocrine organ that could influence fertility through the hormonal secretion of adipokines, which are cytokines involved in various physiological processes [3,4,5]. Those biologically active proteins are considered the main regulators of whole body energy homeostasis [3,6,7,8]. Many reviews have already described and discussed the crucial roles of leptin and adiponectin in different physiological processes including reproduction [9,10,11,12,13,14]. So, here we focused on four novel adipokines named chemerin, visfatin, resistin and apelin that have been also identified and recognised as important regulators of energy metabolism [15,16,17,18]. Moreover, several studies have highlighted their involvement in reproductive functions, in normal or pathological contexts [19,20,21,22]. In this present review, we will discuss the structure of these adipokines and their roles in the male and female reproductive tract in human and animal models, with a discussion of their involvement in several female and male reproductive pathologies including polycystic ovary syndrome and gestational diseases (gestational diabetes mellitus, preeclampsia and intra-uterine growth retardation) and sperm abnormalities and testicular pathologies, respectively.

## 2. Structure of Adipokine Genes and Proteins

### 2.1. Chemerin

**Figure 1:**
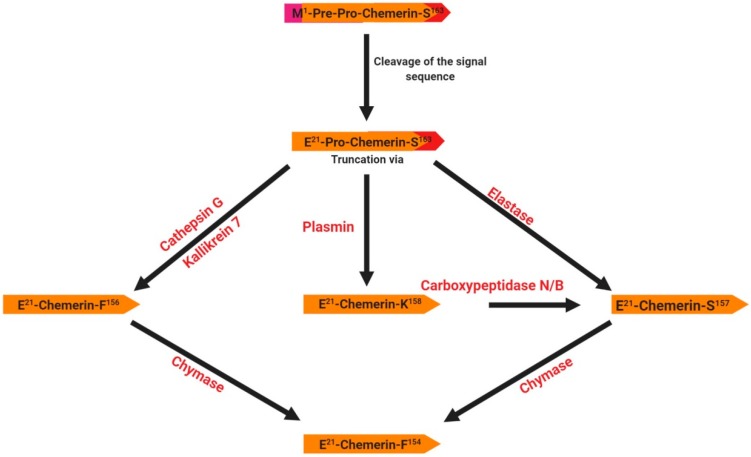
Structure and specific forms of human chemerin. The initial preprochemerin and its different products are processed by different proteases related to inflammation. The signal peptide (purple) is cleaved prior to secretion. Then, the C-terminus is cleaved by different proteases giving several active isoforms such as chemerin F^156^, chemerin S^157^ and chemerin K^158^.

Chemerin was identified in 2003 as the product of the *rarres2* (retinoic acid receptor responder 2) gene, which is located on chromosome 7 in humans and is composed of 5 coding exons with a full length of 1618 bp [23,24]. Expression of this gene is up-regulated by the synthetic retinoid tazarotene and occurs in a wide variety of tissues [23,24]. This gene encodes a 163 amino acid protein (16 kDa) secreted in an inactive form as prochemerin, with the secreted form being 20 amino acids shorter due to the cleavage of the C-terminus by inflammatory and coagulation serine proteases [25] (Figure 1). The cleavage of the signal peptide results in the release of an inactive precursor (chemerin-S163) into the extracellular media. The precursor requires further extracellular C-terminal cleavages at various sites to generate active and deactivated chemerins. For example, the proteolytic cleavage by plasmin, elastase and cathepsin G activates chemerin and generates various isoforms (chemerin-K158, -S157 and -F156, respectively) (Figure 1). Further cleavage of bioactive chemerin by chymase produces chemerin-F154 and terminates its activity [26]. Thus, several isoforms of chemerin have been identified that are dependent on processing by various serine and cysteine proteases (Figure 1). The role of these various forms is still unclear. Chemerin mRNA is detected in a wide range of different tissues in various species including humans, rodents, bovine and poultry [27]. In humans, it is most abundantly expressed in white adipose tissues, the liver and the placenta, and to a lesser extent in brown adipose tissue, the lungs, skeletal muscles, kidneys, ovaries, and the heart [28]. Chemerin is a cytokine described as an important regulator of several physiological processes such as blood pressure control, immune system regulation, angiogenesis and inflammation [26]. It was first discovered in human inflammatory fluids as a potential ligand of an orphan G protein-coupled receptor related to chemokine receptors called ChemR23 or CMKLR1 (Chemokine-Like Receptor 1, cf. paragraph 3.1).

### 2.2. Visfatin

**Figure 2:**
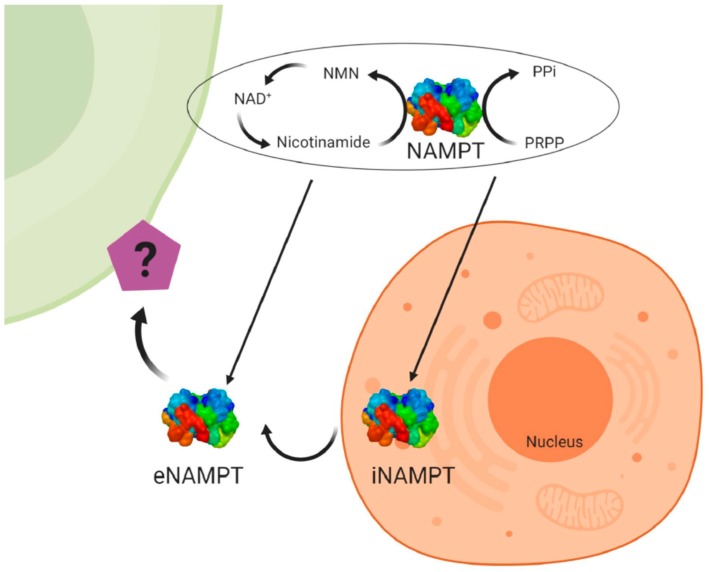
Structure and specific forms of human visfatin. Human visfatin can be found under the intra-cellular form of nicotinamide phosphoribosyltransferase (iNAMPT) having an enzymatic role to produce NAD+ (nicotinamide adenine dinucleotide), and under the extracellular form (eNAMPT) with the same role. Visfatin acts also as a cytokine that could act on target cells. NAMPT catalyzes the reaction between nicotinamide and 5-phosphoribosyl-1-pyrophosphate (PRPP) to yield nicotinamide mononucleotide (NMN), an intermediate in the biosynthesis of NAD+.

Visfatin/NAMPT (nicotinamide phosphoribosyltransferase) was originally cloned in 1994 as a cytokine named pre-B-cell colony enhancing factor (PBEF) from a human peripheral blood lymphocyte cDNA library [29]. The mRNA for *PBEF* is 2.4 kb long and codes for a 52-kDa secreted protein. The 3′ untranslated region is 69% AT and contains multiple TATT motifs. There are two atypical polyadenylation signals, AATAAA, located upstream of the 3′ end. The protein lacks a typical signal sequence for secretion. Human PBEF has ubiquitous expression, although it is predominantly expressed in the human bone marrow, liver, and muscles [29]. In 2001, a study identified the gene *nadV*; its presence allows nicotinamide adenine dinucleotide (NAD)-independent growth of the Gram-negative bacteria *Haemophilus influenza* and *Actinobacillus pleuropneumoniae*. The authors found *NadV* to have significant sequence homology to *PBEF*, thereby suggesting a novel role for PBEF in NAD biosynthesis [30]. Indeed, in 2002 the murine homologue of PBEF was found to be an enzyme catalysing the reaction between nicotinamide and 5-phosphoribosyl-1-pyrophosphate to yield nicotinamide mononucleotide (NMN), an intermediate in the biosynthesis of NAD [31]. The crystal structure of a dimeric PBEF, now called NAMPT, in the presence and absence of NMN further underscores NAMPT as an important enzyme in NAD biosynthesis [32] (Figure 2). At the same time, visfatin has been identified as a cytokine hormone and an enzyme involved in metabolic (obesity, type II diabetes) and immune disorders [33]. In humans, visfatin plasma concentrations are positively correlated with measures of obesity [34]. In mammals, visfatin or NAMPT exists as two forms, the intra- and extracellular forms, iNAMPT and eNAMPT, respectively [35] (Figure 2). In mice, the protein expression of iNAMPT is highest in brown adipose tissue (BAT), and the liver and kidneys, at intermediate levels in the heart, low in white adipose tissue (WAT), and the lungs, spleen, testis, and skeletal muscle, and under detectable levels in the pancreas and brain [35]. While the function of iNAMPT has been firmly established as an NAD biosynthetic enzyme and having an important role in sirtuin activation in mitochondria, the function of eNAMPT is controversial. eNAMPT is released by a number of normal cell types, such as adipocytes, hepatocytes, myocytes, pancreatic cells, neurons and immune cells [35,36]. Moreover, it has been shown that eNAMPT is released under pathological conditions by cancer cells and that it could be used as a marker for cancer development [37,38,39].

### 2.3. Resistin

**Figure 3:**
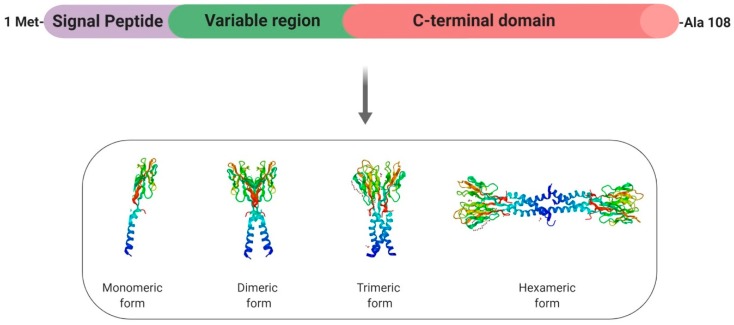
Structure and specific forms of human resistin. Human resistin is composed by a signal peptide (purple), a variable region (green) and a C-terminal domain. Resistin can be found under the monomeric form and can form dimeric and trimeric proteins thanks to disulfide bridges. Then, disulfide and non-disulfide bridges can be involved in the formation of the hexameric protein.

Resistin (Retn) is a pro-inflammatory adipokine that was first identified in mice about 20 years ago, where it was identified as “adipose-tissue-specific secretory factor” (ADSF) [40], “found in the inflammatory zone 3” (FIZZ3) [41] and eventually renamed “resistin” (or “resistance to insulin”) due to its ability to resist the action of insulin [42]. The gene coding for human resistin is located on chromosome 19p13.2 and spans 1369 bp, with three introns and four exons [43]. Resistin is a 108-amino acid propeptide, which includes a signal peptide, a variable region, and a conserved C-terminus [42] (Figure 3). Resistin (12.5 kDa) circulates in human blood as a dimeric protein consisting of two 92-amino acid polypeptides that are linked by a disulphide bridge and forms high- and low-molecular weight complexes (Figure 3). Indeed, a common feature of resistin is the existence of a motif (10–11 cysteine-rich) at the carboxyl terminus that could support the globular domain of the resistin monomer via the formation of 5 disulphide bridges [44,45] (Figure 3). Disulphide and non-disulphide bonds also play an important role in the formation of dimer, trimer, and hexamer forms of circulating resistin. In mice, the *Retn* gene is almost exclusively expressed in white adipocytes and blood cells [42], whereas peripheral blood mononuclear cells (PBMCs), macrophages, and bone marrow cells are the primary source of circulating resistin in humans [46]. In rodents, resistin represents a clear pathogenic factor in the severity of insulin resistance (IR) [42]. However, in humans, despite initially being proposed as the potential link between obesity and diabetes [47], this adipokine does not represent a major determinant of IR. Indeed, plasma human resistin seems to be correlated wth IR as a consequence of obesity itself rather than as an independent causative factor [47].

### 2.4. Apelin

**Figure 4:**
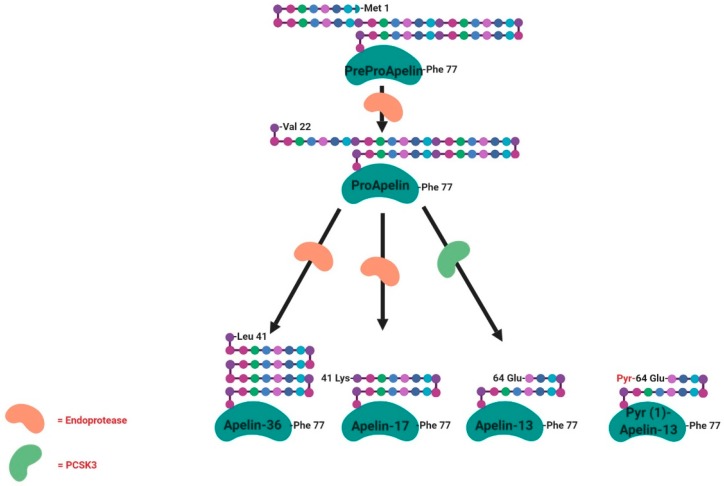
Structure and specific forms of human apelin. Human apelin is first of all found under the preproapelin form (77 amino acids) that will be cleaved by endopeptidases acting on basic amino-acid-rich regions giving the proapelin (55 amino-acids) and then the other tissue-dependent active isoforms (36, 17, 13 and Pyr-13 amino-acids). PCSK3 (proprotein convertase subtilisin/kexin 3) is involved in the cleavage of proapelin to apelin-13.

APJ, now known as apelin receptor, was considered as an orphan G protein-coupled receptor. In 1998, a Japanese team purified a peptide that is able to bind the APJ orphan receptor, named apelin, from bovine stomach extracts [48]. Apelin is widely expressed in various types of tissues and organs such as the central nervous system and peripheral tissues, including the hypothalamus, adipose tissue, skeletal muscle, digestive system and the ovary [49,50,51,52]. The human apelin gene is found on chromosome Xq25-q26.1. The apelin gene sequence contains an intron of around 6kb in length with recognised intron/exon boundaries interrupting the ORF (Open Reading Frame) at the position encoding Gly22. Similar to the rat preproapelin cDNA, the reported start codon for human preproapelin does not appear to conform to the Kozak consensus sequence, again due to the presence of an adenosine immediately following the start codon. The cDNA of apelin encodes a 77-amino-acid preproprotein (Figure 4). The N-terminal sections of bovine and human proteins are rich in hydrophobic amino acids, indicating that these represent secretory signal sequences. The amino acid sequence of the isolated bovine peptide corresponds to the deduced sequence of the bovine preproprotein from position 42 to position 58. These results suggest that apelin is one of the processing products derived from the C-terminal portion of the preproprotein [48]. To date, the main active forms of apelin are apelin-13, -17 and 36 and the pyroglutaminated isoform of apelin-13 (Pyr(1)-apelin 13), which is characterised by a higher resistance to degradation [53] (Figure 4). In the heart, the described predominant form is the Pyr(1)-apelin-13 [54]. Pyr(1)-apelin-13, apelin-13 and apelin-36 have comparable efficacy and potency in human cardiovascular tissues [54], whereas apelin-17 appears to be the most efficient in promoting apelin receptor internalisation [55]. The rat, bovine and human preproapelin sequences for the last 23 C-terminal amino acids share a sequence homology of 100% [48].

## 3. Adipokines Receptors and Signalling Pathways

### 3.1. Chemerin

**Figure 5:**
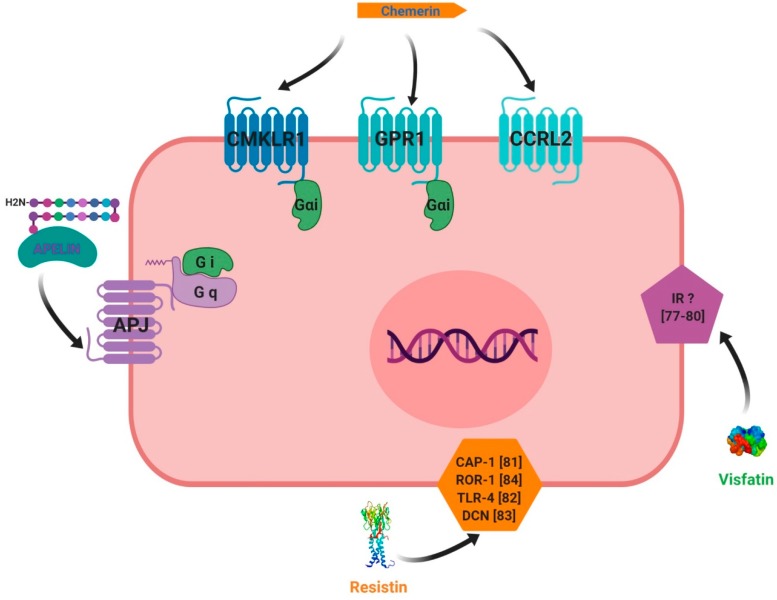
Receptors used by chemerin, visfatin, resistin and apelin for their signaling pathways. chemerin can bind three different receptors that are CMKLR1, GPR1 and CCRL2. CMKLR1 and GPR1 are coupled with intracellular Gαi proteins. The last receptor doesn’t have any active signaling pathway identified until now suggesting a putative role of co-receptor for CCRL2. Apelin has its own receptor called APJ coupled with intracellular Gq and Gαi proteins. Resistin could bind receptors such as CAP-1, ROR-1 and TLR-4 but it has to be confirmed. No receptor for visfatin has been identified until now.

The first chemerin receptor identified was CMKLR1, originally named ChemR23. It has been described as an orphan G protein-coupled receptor and was cloned to identify novel chemotactic factor receptors [56]. Subsequently, it has been found to be expressed in monocyte-derived dendritic cells and macrophages and as a co-receptor for SIV (Simian Immunodeficiency Virus) and some primary HIV-1 (Human Immunodeficiency Virus-1) strains, and is known as ChemR23 [57]. A second receptor, named LPS (LipoPolySaccharide) inducible C-C chemokine receptor-related gene (L-CCR), has been discovered in mouse macrophage activation [58] and its orthologue in human, firstly named as Human Chemokine Receptor (HCR), have been identified in a human neutrophil cDNA library [59]. Firstly, this receptor was considered as an orphan receptor. A third receptor named G Protein Receptor 1 (GPR1), has also been identified as an orphan receptor in humans and rodents, and is involved in peptide transmission in brain functioning [60]. Also, besides CMKLR1, orphan receptors GPR1 and CCRL2 have been identified by in vitro assays as spare receptors for chemerin (Figure 5, [61,62]. Those three receptors are protein G-coupled receptors with seven-transmembrane domains. The expression of CMKLR1 mRNA was detected in a wide variety of tissues such as haematopoietic tissues [24,56,63], adipocytes [64], endothelial cells [65], osteoclasts [66] and ovarian cells [67]. CCRL2 has been found to be expressed in cell types such as macrophages [68], mast cells [62], lung epithelial cells [69] and the ovary [67]. Contrary to CMKLR1 and CCRL2, GPR1 expression has not been found in immune cells but has been reported in central nervous system cells [60,70], murine brown adipose tissue, white adipose tissue, and skeletal muscle. GPR1 is mainly expressed in vascular cells in white adipose tissue [16]. Chemerin binding to CMKLR1 enhances leukocyte chemotaxis [71]. Chemerin binding to CCRL2 does not mediate cell signalling, but might present chemerin to nearby CMKLR1-positive cells to promote its function and play a key role in immune responses, inflammation, and other physiological processes (Figure 5, [62]. GPR1 is an active receptor of chemerin and could regulate glucose homeostasis in the development of obesity because glucose intolerance was found to be increased in Gpr1-knockout mice fed a high-fat diet compared to wild-type (WT) mice. Within the mice ovary, it has been shown that chemerin/GPR1 signalling regulates progesterone secretion during the processes of follicular development, corpus luteum formation, and PGF2α-induced luteolysis [72].

Chemerin binding to CMKLR1 activates the three Gαi subtypes and the two Gαo isoforms (Figure 5). Chemerin stimulates the increase in intracellular calcium and a decrease of cyclic AMP in CMKLR1-expressing CHO cells that are dependent on Gαi signalling [24]. It further uses the RhoA- and Rho-associated protein kinase-dependent pathway downstream of GPR1 and CMKLR1 to activate the transcriptional regulator serum-response factor [73]. Mitogen-activated protein kinase ERK1/2 is phosphorylated upon chemerin treatment in various cells, including endothelial cells, adipocytes, and skeletal muscle cells. The activation of ERK1/2 at low, but not high, chemerin concentrations was described in adipocytes [65,74]. Chemerin further activated p38 mitogen-activated protein kinase, Akt and phosphoinositide 3-kinase [26]. Short periods of incubation with chemerin were shown to activate Akt in hepatocytes, whereas prolonged treatment of up to two hours led to a decline in phosphorylated Akt in these cells [75]. The same study reports that chemerin binding weakened the association of phosphatase and tensin homolog (PTEN) with CMKLR1. This enhanced the activity of PTEN and subsequently led to decreased Akt phosphorylation. The nuclear factor kappa B (NFkB) pathway is activated by chemerin in skeletal muscles cells [76]. Chemerin thus activates various signalling pathways, with effects that are dependent on incubation time and dose.

### 3.2. Visfatin

The receptor of visfatin is still unknown (Figure 5) and the exact cellular mechanism of extracellular visfatin (eNAMPT) remains unclear, even though various authors have implicated intracellular insulin receptor signalling pathways in the action of visfatin [77,78,79,80].

### 3.3. Resistin

Like visfatin, the receptor of resistin remains unknown and the molecular mechanism of resistin action is unclear. However, recent reports have suggested potential receptors for resistin (Figure 5). In humans, there are two putative resistin receptors: adenylyl cyclase-associated protein 1 (CAP 1) [81] and toll-like receptor 4 (TLR4) [82]. Studies have involved the use of an isoform of decorin (DCN) [83], as this is supposed to be a receptor of resistin. Moreover, mouse receptor tyrosine kinase-like orphan receptor 1 (ROR1) has been identified as a putative receptor for resistin [84]. It is well known that resistin activates signalling pathways in different tissues such as Akt, MAPK (Mitogen-Activated Protein Kinases, ERK1/2 and p38), Stat-3 (signal transducer and activator of transcription 3) and PPAR gamma (PPARγ).

### 3.4. Apelin

Apelin receptor (APJ) was first identified in 1993 as a class A (rhodopsin-like) orphan G protein-coupled receptor which shows high homology with the angiotensin II (AngII) receptor [85]. The gene encoding APJ is intronless and is known as *APLNR* in humans. The *APLNR* gene encodes a 380-amino acid protein and is located on chromosome 11q12 [85]. The promoter of the rat APLNR gene does not include a TATA box, but contains a potential CAAT box at −1257 bp and a number of activator protein 1 and specificity protein 1 (Sp1) motifs [86]. There are two transcriptional start sites at −247 and −210 bp [86]. The protein structure of APJ is typical of a GPCR (G protein-coupled receptor), containing seven hydrophobic transmembrane domains, with consensus sites for phosphorylation by protein kinase A (PKA), palmitoylation and glycosylation (Figure 5, [85]. The apelin system is able to activate a high number of signalling pathways through various G proteins. Apelin-13 and apelin-36 activate the phosphorylation of ERK1/2 in Chinese hamster ovary (CHO) cells stably expressing mouse APJ [87]. The phosphorylation, and thus activation, of Akt has been shown to be a downstream effector of apelin signalling; this was first shown to occur via a PTX-sensitive G-protein and PKC [87]. The same study showed that Apelin induces the dual phosphorylation of the S6 ribosomal protein kinase (p70S6K) in human umbilical vein endothelial cells (HUVECs), where apelin promotes cell proliferation via PTX-sensitive, ERK1/2-, mammalian target of rapamycin (mTOR)-, and Akt-dependent intracellular cascades. In the absence of ligands, the apelin receptor is also able to heterodimerise with other GPCRs and activate signalling pathways.

## 4. Adipokines and Reproductive Functions at the Hypothalamo-Pituitary Level in Non-Pathological Conditions

### 4.1. Chemerin

**Figure 6:**
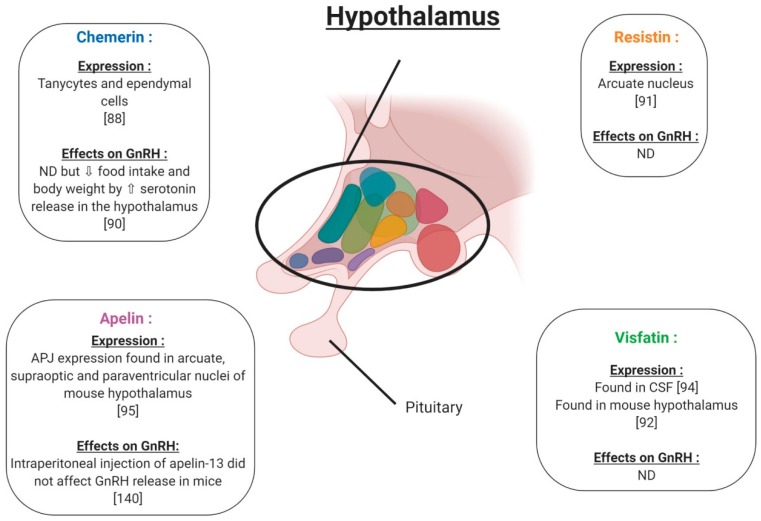
Expression and effects on GnRH (Gonadotropin-Releasing Hormone) release of chemerin, visfatin, resistin and apelin in hypothalamus. ⇧ Increase/stimulation. ⇩ Decrease/inhibition. ND: not determined. CSF: cerebrospinal fluid.

**Figure 7:**
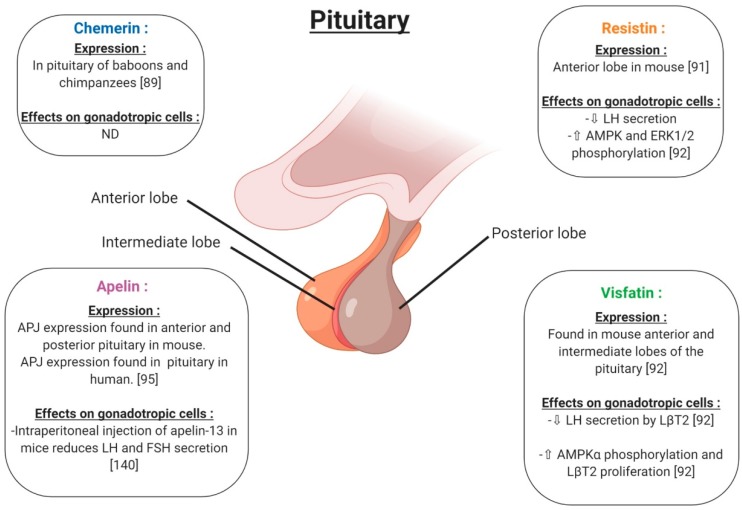
Expression and effects on gonadotropic cells of chemerin, visfatin, resistin and apelin in pituitary. ⇧ Increase/stimulation. ⇩ Decrease/inhibition. ND: not determined.

In the hypothalamus, the expression of chemerin and its receptors was observed within the tanycytes and ependymal cells (Figure 6), with increased expression reported for long (LD) versus short (SD) photoperiods, pointing to a physiological role. [88]. In the pituitary, *RARRES2* gene expression has been found in baboons and chimpanzees [89] (Figure 7). Furthermore, in rats, chemerin administration (8 and 16 μg/kg) decreased both food intake and body weight compared to vehicle, possibly associated with a significant increase in serotonin synthesis and release, in the hypothalamus [90]. These results suggest an important role of chemerin in hypothalamus–pituitary function especially in feeding behaviour; however, interactions between chemerin and the central nervous system involved in reproduction need further investigation. No in vivo or in vitro studies have investigated the effect of chemerin on pituitary cells.

### 4.2. Resistin

In rodents, resistin is expressed in the hypothalamus in a region responsible for energy balance [91] (Figure 6). Maillard et al. (2017) showed staining by immunohistochemistry of resistin in the anterior lobe of the mouse pituitary (Figure 7). Resistin 1 and 10 ng/mL decreased LH (Luteinizing Hormone) secreted by LβT2 cells. Furthermore, a 0.01; 0.1 and 1 ng/mL resistin concentration significantly decreased mouse pituitary cell LH secretion, but 10 ng/mL resistin did not significantly affect LH release [92] (Figure 7). Thus, the effect of resistin on pituitary cells seems to be dependent on the concentration. In LβT2 mouse cells, resistin (1 ng/mL) increased phosphorylation in the AMPK and ERK1/2 signalling pathways (Figure 7). Furthermore, resistin has been shown to be regulated by gonadotrophins from the pituitary [93]. All of these data suggest a potential role of resistin in the hypothalamo-pituitary axis.

### 4.3. Visfatin

Visfatin has been found in the cerebrospinal fluid but its origin is not known [94]. Visfatin is present in the mouse brain, hypothalamic area (Figure 6) and pituitary, as well as in LβT2 mouse gonadotrophin cells [92]. By immunohistochemistry, visfatin was localised in the anterior and intermediate lobes of the pituitary and seems to be co-localised with βLH (Figure 7). In LβT2 cells, LH secretion was decreased by visfatin stimulation from 0.1 to 10 ng/mL, whereas visfatin did not affect LH secretion in the primary mouse pituitary cells [92]. Thus, visfatin could play a regulatory role at the hypothalamo-pituitary level.

### 4.4. Apelin

Apelin receptor (APJ) has been characterised in the mouse central nervous system by immunohistochemistry (Figure 6). APJ was localised in hypothalamic nuclei, such as arcuate, supraoptic and paraventricular nuclei, and in the anterior pituitary, implying potential roles in the control of reproduction [95]. These brain regions are known to be the central control points of energy utilisation and reproductive behaviour. The intraperitoneal injection of apelin-13 in mice reduced LH, FSH (Follicle Stimulating Hormone) and testosterone serum levels and showed negative effects on the reproductive function (Figure 7). However, in these mice, no measurement of GnRH release was performed. Based on these results, the authors suggested that the inhibitory effect of apelin on testosterone levels was due to a direct action of apelin on gonadotropic cells [96,97,98].

## 5. Adipokines and Reproductive Functions at the Gonad Level in Non-Pathological Conditions

### 5.1. Ovary

#### 5.1.1. Chemerin

**Figure 8:**
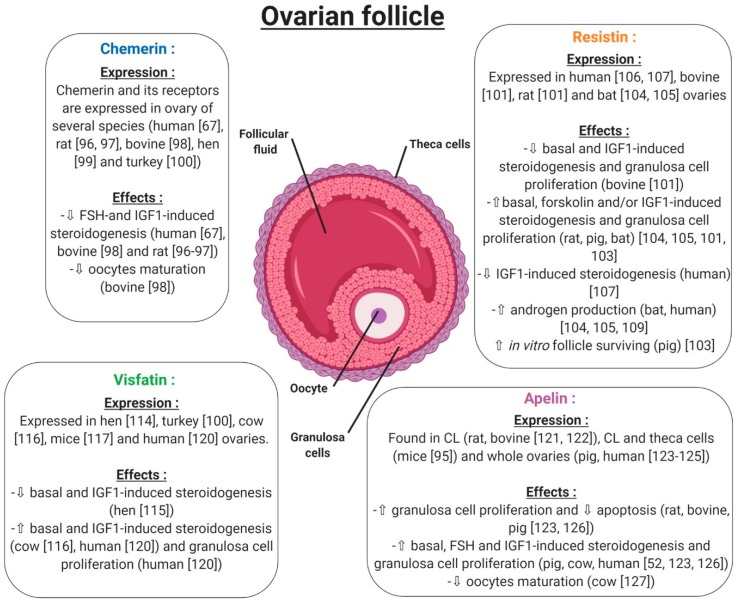
Expression and effects of chemerin, visfatin, resistin and apelin on the ovarian follicle. ⇧ Increase/stimulation. ⇩ Decrease/inhibition.

Under normal physiological conditions, chemerin and CMKLR1 have been shown to be expressed in the mouse ovary [74]. Another study went further, demonstrating that the chemerin system is expressed within the human ovary at mRNA and protein levels, but also has a functional role in ovarian physiology (Figure 8). This study showed that chemerin and its receptor CMKLR1 are present and active in human granulosa cells. Chemerin reduces IGF-1 (Insulin Like Growth Factor 1)-induced steroidogenesis and cell proliferation through a decrease in the activation of IGF-1R signalling pathways in primary human granulosa cells (hGCs) [67]. Chemerin also inhibits FSH-induced mRNA and protein expression of aromatase and p450scc in rodent granulosa cells [99,100] (Figure 8). In bovine species, chemerin and its three receptors CMKLR1, GPR1 and CCRL2 are expressed within the ovary and recombinant chemerin decreases in vitro steroidogenesis and cholesterol synthesis at the basal level as well as after IGF-1 and/or FSH induction [101] (Figure 8). This down-regulation is associated with a reduction in protein levels of STAR (Steroid Acute Regulatory protein), cytochrome P450 family 19 subfamilies A member 1, and 3-Hydroxy-3-Methylglutaryl-CoA Reductase and the phosphorylation of MAPK ERK1/2, which is dependent on CMKLR1. Moreover, human recombinant chemerin also inhibits bovine oocyte maturation at the germinal vesicle stage and decreases MAPK ERK1/2 phosphorylation in oocytes and cumulus cells [101] (Figure 8). In avian species, it has also been demonstrated that chemerin and its three receptors are expressed in the hen ovary and that chemerin plasma concentration is positively correlated with egg hatchability [102]. Chemerin and its receptors are also expressed in turkeys, more particularly in the theca and granulosa cells, with higher levels in theca cells. Moreover, this study showed that chemerin plasma concentrations decreased during the laying period [103]. All of these data suggest that chemerin could be a key hormone linking reproductive and metabolic functions.

#### 5.1.2. Resistin

Resistin has been shown to be expressed within the ovaries of rodent and bovine species [104]. More precisely, resistin is expressed by theca cells, luteinised granulosa cells of the corpus luteum and oocytes, but its expression is very weak in granulosa cells. By contrast, in bovine species, resistin is expressed by granulosa cells of growing follicles, with its expression decreasing in the corpus luteum and being almost undetectable in oocytes (Figure 8). Furthermore, in these cells, the authors observed that recombinant resistin can modulate steroidogenesis and proliferation in a basal state or in response to IGF-1 in vitro [104] (Figure 8). Another study showed that resistin inhibits steroidogenesis of undifferentiated (small follicles) granulosa cells and mitogenesis of differentiated (large follicle) granulosa cells [105]. These results highlighted a huge species difference between rat and bovine species in terms of resistin expression and its effects [104]. In a porcine model, resistin is expressed by theca cells, but also and more abundantly in granulosa cells of growing follicles (Figure 8). In these cells, resistin supports the survival of in vitro cultured porcine follicles (Figure 8). It also has a positive effect on steroidogenesis and mitosis on ovarian cells [106]. In the greater Asiatic yellow bat (*Scotophilus heathii*), resistin is expressed in the ovary, more precisely by the theca cells, as in rats, with an increase in expression around the ovulation period. Moreover, recombinant resistin alone on cultured bat granulosa cells preferentially stimulates progesterone secretion. Furthermore, resistin increases androgenic action in the ovary [107,108].

In humans, resistin expression has been demonstrated in luteinised granulosa cells recovered after follicle punction in a programme of in vitro fertilisation [109,110]. In 2009, a study demonstrated that ovarian hormones did not have any effects on resistin concentrations in serum [111]. However, resistin itself influences in vitro ovarian functions. Indeed, Munir et al. demonstrated that resistin enhances mRNA expression and the activity of 17alpha-hydroxylase in human theca cells in the presence of forskolin and insulin [112] (Figure 8). This finding confirms the results obtained in rat testes, where resistin increases testosterone secretion in a dose-dependent manner [93]. In contrast, Reverchon et al. showed that resistin decreases IGF-1-induced steroidogenesis in primary human granulosa cells. These effects were confirmed by Messini et al. in response to FSH [113]. Despite its negative effect on the regulation of ovarian steroidogenesis [110,114] (Figure 8), resistin does not seem to play a key role in human ovarian physiology. Indeed, its levels in follicular fluid have been repeatedly found to be lower than in plasma [110,115], suggesting that human granulosa cells, while expressing resistin protein [110], are unlikely to secrete it into follicular fluid or the circulation. Varnagy et al. showed that resistin levels in follicular fluid were a positive predictor of oocyte and embryo number, indicating a beneficial effect of this adipokine on the outcome of in vitro fertilisation procedures [116]. However, this finding has not been confirmed by other authors, who concluded that resistin cannot play a significant role in the maturation and development of oocytes [115,117]. This discrepancy could be explained by the number of patients and also by the experimental design.

#### 5.1.3. Visfatin

In the chicken model, this adipokine is widely expressed in a lot of tissues, in a sex-dependent manner (Figure 8). It has been found in hen ovaries at both the mRNA and protein levels [118], and an in vitro study has shown that visfatin inhibits progesterone production in granulosa cells through STAR and HSD3B at basal level or after IGF-1 stimulation [119]. Still in birds, visfatin is expressed mainly by theca cells and more weakly by granulosa cells of turkey follicles. During a laying cycle, its concentration in serum significantly decreases in the same model [103]. In cows, visfatin is expressed within the ovary in theca, granulosa, cumulus cells and oocytes. Contrary to the chicken model, visfatin increases in vitro steroidogenesis and potentialises IGF-1 effects by increasing STAR and HSD3B expression, as well as E2 and P4 secretion [120] (Figure 8). In mice, visfatin is also expressed within the ovary and more precisely in stromal cells, endothelial cells, granulosa cells and cumulus cells. Its main production comes from granulosa cells and increases during follicular growth. The administration of visfatin during ovulation induction in aged female mice improves the developmental competency of oocytes [121] (Figure 8). Furthermore, Shen et al. found a significant positive correlation between visfatin concentration in follicular fluid and the number of retrieved oocytes in women [122], confirming a possible positive role of this adipokine in female reproductive function. However, visfatin concentration in follicular fluid has been shown to be similar [122] or lower [123] than in serum, and no correlation was found between visfatin plasma and follicular fluid levels [122]. Hence, circulating visfatin concentration does not seem to contribute significantly to visfatin concentration in follicular fluid [122] which could, therefore, be independently and differently regulated at the ovarian level. In humans, visfatin is expressed in granulosa cells, but also in cumulus cells and oocytes, and less abundantly in theca cells. Moreover, recombinant human visfatin increases cell proliferation and E2 and P4 production by luteinized human granulosa cells [124]. Thus, as in bovine species and mice, visfatin has a positive effect on steroidogenesis in human granulosa cells [124].

#### 5.1.4. Apelin

Apelin and APJ have been detected by in situ hybridisation in the corpus luteum (CL) of the rat ovary [125]. In mice, the apelin system is found in the corpus luteum but also in theca cells of follicles, showing a species difference between mice and rats [95]. In bovine species, this adipokine is also expressed in the early and mid-luteal stages of the corpus luteum to then decline during the regression phase. More precisely, apelin was localised in the smooth muscle cells of intraluteal arterioles, and responded to PGF2-alpha at the periphery of CL in cows [126]. In porcine species, the expression of apelin and APJ increased with ovarian follicular growth [127]. In humans, apelin and its receptor are expressed by granulosa, cumulus, and theca cells with a putative weak expression in oocytes (Figure 8). Even if plasma apelin levels are largely dependent on the assay, apelin concentration in follicular fluid seems to be higher than in plasma [128,129]. It can thus be speculated that follicular apelin is partly derived by granulosa cell production and regulates the function of granulosa cells in a paracrine and/or autocrine manner. Indeed, although mice lacking apelin or APJ genes are viable and fertile [52], some in vitro evidence suggests a potential role of apelin in the control of ovarian function. Indeed, it enhances progesterone and oestradiol secretion in human and porcine granulosa cells [52,96,127] (Figure 8). In primary human granulosa cells, Apelin-13 and Apelin-17 isoforms are both able to increase basal and IGF-1-induced progesterone and oestradiol secretion, which was associated with an increase in HSD3B protein concentration and AKT and MAPK ERK1/2 phosphorylation [52]. Apelin also improves rat, bovine and porcine granulosa cell proliferation [127], and seems to be involved in the regulation of bovine corpus luteum luteolysis processes [126] and oocyte maturation [97]. Notably, apelin has been suggested to be implicated in bovine follicular atresia [96] and, in different animal species, both the mRNA and protein levels of apelin and APJ changed during follicular growth with the highest expression in large follicles [127]. In bovine species, apelin exerts a negative effect on in vitro oocyte maturation through blocking of the meiotic progression at the germinal vesicle stage [97] (Figure 8).

**Figure 9:**
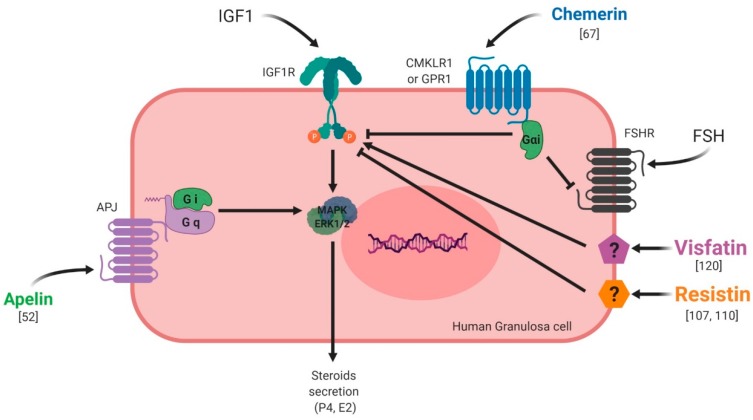
Effects of chemerin, visfatin, resistin and apelin on steroidogenesis in vitro in primary human granulosa cells. Chemerin and resistin are known to inhibit IGF-1 (Insulin Like Growth Factor 1)-induced steroidogenesis whereas visfatin and apelin exert stimulatory effects.

In summary, chemerin and the apelin system, resistin and visfatin are expressed in the ovary of various species. The effects of these adipokines mainly studied in vitro are dependent on the species but also on the dose used, experimental design and probably on the methodological uncertainties. In primary human granulosa cells, chemerin and resistin inhibit in vitro steroidogenesis induced by IGF-1, whereas visfatin and apelin improve it (Figure 9).

### 5.2. Testis

Recent reports have pointed to an emerging role of adipokine in male reproductive functions. Indeed, it has been shown that obesity is positively correlated with defective spermatogenesis, especially in developed countries where semen quality is seriously lowered. Furthermore, obesity is associated with decreased testosterone concentrations and sperm motility [98]; however, the action by which testosterone production is reduced remains unclear. The link between obesity, adipokine and male fertility problems needs to be elucidated. The effect of adiponectin and leptin in male fertility has already been described [19]. In this section, we will focus on the role of other novel adipokines (chemerin, apelin, visfatin, resistin) in male reproductive functions.

#### 5.2.1. Chemerin

**Figure 10:**
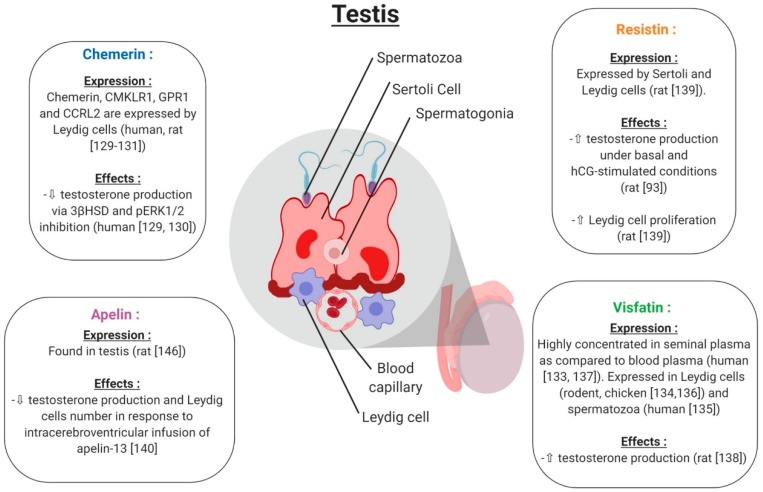
Expression and effects of chemerin, visfatin, resistin and apelin on testicular function. ⇧ Increase/stimulation. ⇩ Decrease/inhibition.

Chemerin and its three receptors CMKLR1, GPR1 and CCRL2 are expressed in rat and human male reproductive tracts. [130,131]. Chemerin, CMKLR1 and GPR1 proteins are specifically localised in Leydig cells of the human and rat testis [132] (Figure 10). In rat primary Leydig cell cultures, chemerin suppressed the testosterone production induced by human chorionic gonadotropin (hGC, [133]) (Figure 10). This is associated with an inhibition of gene and protein expression of the 3β-hydroxysteroid dehydrogenase and MAPK ERK1/2 phosphorylation [133]. The C-20 chemerin derived peptide presents similar effects to chemerin, suggesting that this peptide can present similar functions as the different peptide forms of chemerin [130]. In mice deleted for CMKLR1 (CMKLR1−/−), the plasma testosterone level is lower compared to in wild-type animals [134]. Furthermore, in cultured Leydig cells isolated from CMKLR1−/− mice, gene expression of *HSD3B*, *STAR*, *P450scc*, *Sf1*, *Gata4* and *Insl3* was significantly decreased compared to wild-type mice. All of these data suggest that chemerin plays an important role in male steroidogenesis, as in females. In humans, chemerin is present in seminal plasma at lower concentrations than in human blood plasma [135]. Obesity is associated with reduced sperm motility and lower testosterone serum levels, and it is known that chemerin secretion is increased in the case of obesity, suggesting that chemerin could have a negative effect on sperm motility/maturation in the epididymis through direct effects on spermatozoa or indirect effects during epidydimal maturation.

#### 5.2.2. Visfatin

Visfatin is present in rodent and human testes [136,137]. It is also expressed in pre-pubertal and adult chicken testes, and more particularly in the nucleus of sertoli cells, and Leydig cells [138] (Figure 10). In humans, visfatin concentrations in seminal plasma is higher than in blood plasma [139] suggesting local production or by the male annex glands. Moreover, visfatin is present in the human spermatozoa and is regulated in a maturation-dependent manner [137]. In rats, visfatin increased testosterone production through Ras1 kinase from in vitro cultured Leydig cells [140] (Figure 10). Moreover, Jeremy et al. showed that visfatin protein expression in the rodent testis is decreased by a D-galactose treatment which induces aging [136]. The visfatin expression is specifically decreased in Leydig cells and decreases serum testosterone levels. These data suggest an important role of visfatin in testicular aging by regulating spermatogenesis and steroidogenesis [136].

#### 5.2.3. Resistin

Resistin has been found to be expressed in the testis and seminiferous tubules of rats, and especially in Sertoli and Leydig cells. Resistin is equally expressed in mouse Leydig cell lines (MA-10 and TM3) [141]. In rat Leydig cells, in vitro stimulation with resistin increased both basal and human chorionic gonadotropin (hCG)-stimulated testosterone production [93] (Figure 10). Roumaud et al. cited that exposure to a low resistin concentration (10 ng/mL = obesity physiological concentration) increases Leydig cell proliferation [141]. These data suggest that resistin could positively regulate Leydig cell steroidogenesis and proliferation.

#### 5.2.4. Apelin

Apelin is found in the rat testis [142]. Only one study showed that the intracerebroventricular infusion of apelin-13 in male rats significantly reduced serum testosterone levels compared to the control group [143] (Figure 10). Histological analyses demonstrated a reduction in the number of Leydig cells, suggesting that apelin may play a role in the central regulation and decrease testosterone release by suppressing LH secretion (Figure 10).

## 6. Adipokines and Pregnancy

### 6.1. Adipokines and Uterus

Chemerin was first identified in normal human myometrial cells and fibroidic cells by microarray and real-time quantitative polymerase chain reaction (RT–qPCR) [144]. Later, it was demonstrated that chemerin is expressed in human primary cultures of stromal cells and extravillous trophoblast cells from pregnant women. Moreover, data indicate that chemerin is up-regulated during decidualisation and might contribute to natural killer (NK) cell accumulation and vascular remodelling during early pregnancy [145]. In the human and rat myometrium, visfatin had similar dose-dependent effects on the inhibition of both spontaneous and oxytocin-induced contractions of pregnant rat and human myometrial tissue in vitro [146]. In the mouse model, visfatin is also expressed by the uterus and its expression varies during the sexual cycle. Indeed, it appeared that E2 and P4 regulate visfatin expression explaining variations during sexual cycle expression in mice uterus [113].

Only one study has demonstrated that resistin is expressed within the uterus, by showing that this adipokine is expressed in the ovine uterus and that its expression varies according to the nutritional status of the animal, an observation that strengthens the link between nutritional status and reproductive activity [147]. First evidence of apelin expression in the uterus appeared in 2001 in a study showing the purification of the apelin-36 isoform by gel filtration chromatography from uterine tissue extracts from rats [148]. As in rats, apelin and its receptor appear to be expressed in the mouse uterus [95]. In humans, an in vitro study brought evidence that apelin had inhibitory effects on uterine contractility by down-regulating spontaneous and oxytocin-induced contractions in the human myometrium [149]. Similar results have since been found in rats [150].

### 6.2. Adipokines and Placenta

Acting as a chemoattractant, chemerin has been hypothesised to play a role in placentation. Indeed, this adipokine is produced by the rat placenta during gestation and has also been found to be expressed in human placenta. Chemerin plays a role in placentation by regulating NK cell accumulation and endothelial cell morphogenesis during early pregnancy, along with the expression of this adipokine in stromal cells and extravillous trophoblast cells; however, this expression remains low compared to that in the liver and adipose tissues [145,151]. It has also been found to be expressed within the umbilical cord, playing a protective role by regulating umbilical vein endothelial cell-induced nitric oxide signalling in preeclampsia [152]. Finally, the chemerin/GPR1 system has been found to be expressed in mice and human placenta with a putative role as a feedback mechanism that could regulate the carbohydrate balance during pregnancy [153].

Visfatin has been immunolocalised throughout gestation in the amniotic epithelium and mesenchymal cells as well as the chorionic cytotrophoblast and parietal decidua. This adipokine is constitutively expressed by the foetal membranes during pregnancy. It increased the expression of IL-6 and IL-8 and may be important in both normal spontaneous labour and infection-induced preterm labour [154]. It has also been linked with the initiation of normal labour with a role at the end of the signalling cascade. Indeed, a previous study demonstrated that visfatin activates pro-inflammatory cytokine release and phospholipid metabolism in the human placenta via activation of the NF-κB pathway, leading to normal labour [155,156]. Its production has also been linked to the equilibrium and homeostasis of amniotic fluid by regulating its reabsorption by the amniotic fluid through stimulation of VEGFR2 ((Vascular endothelial growth factor receptor 2) expression in the placenta [157]. In vitro, the PPAR-γ signalling pathway has been linked to the regulation of visfatin by IL-6 in BeWo (Placental cell line cell), providing a novel insight into the roles of visfatin in trophoblastic cells [158].

In a rat model, resistin expression has been demonstrated in the placenta under normal conditions [159]. First evidence of its expression in the human placenta was provided by Yura et al. in 2003 [160] with a study showing that this adipokine is produced by the placenta with a maximum at full-term pregnancy. Resistin mRNA and protein are also localised to the syncytiotrophoblast and EVTs in early gestation and the syncytiotrophoblast in late gestation [160]. Maternal serum levels in the first and second trimesters are relatively constant and comparable to values in non-pregnant women. However, resistin levels and placental mRNA expression are increased by the third trimester [161]. Because adipose resistin expression remains unchanged during pregnancy, placental production is likely a major source of resistin in the maternal circulation. Such changes in resistin levels could contribute to the decrease in insulin sensitivity during the latter half of pregnancy, which is beneficial for the rapid growth of the foetus [162]. In the human placenta, resistin could affect glucose-uptake, presumably by decreasing the cell surface glucose transporter [163].

The apelin system is also expressed within the human placenta under normal conditions [164]. In rats, apelin expression is lower than in the brain, with a localisation of the protein in the perivascular smooth muscle [165]. In the same species, in cases of induced hypertension by nitric oxide treatment, the apelin receptor, but not apelin itself, is up-regulated in the placenta. The authors of this study concluded that this suggests that the apelinergic system may control foetal growth and cardiovascular functions in utero [133]. In humans again, labour down-regulates apelin expression in foetal membranes. Furthermore, a role of apelin in the regulation of pro-inflammatory and pro-labour mediators, like interleukin (IL)-1β-induced IL-6, IL-8 release and cyclooxygenase-2, is suggested in foetal membranes [166]. Moreover, apelin controls foetal and neonatal glucose homeostasis and is altered by foetal growth restriction induced by maternal under-nutrition [167]. Finally, apelin seems to be implicated in trophoblastic amino acid transport by stimulating amino acid uptake by the placenta [168].

## 7. Adipokines and Female Reproductive Pathologies

### 7.1. Polycystic Ovary Syndrome

Polycystic ovary syndrome (PCOS) is a very common endocrinopathy affecting 6% to 13% of women of reproductive age and one of the leading causes of female poor fertility [169]. According to recommendations in 2018 from the international evidence-based guidelines, its diagnosis requires the presence of at least two of the following criteria: oligo/anovulation, hyperandrogenism and polycystic ovary morphology on ultrasound (corresponding to a follicle number per ovary >20 and/or an ovarian volume >10 mL on either ovary) [170]. Despite its high prevalence and relevant impact on female health, the aetiology of PCOS, and notably the causal relationship between reproductive and metabolic features, has not yet been fully elucidated. Typically, PCOS is associated with visceral obesity [171] and an original adipose tissue dysfunction, possibly due to an in utero androgen hyperexposure, which is supposed to play a key role in determining both insulin-resistance (IR) and altered androgen metabolism characterising this syndrome [172]. Indeed, 50 to 90% of PCOS females have IR to a significantly greater extent than age- and body mass index (BMI)-matched control women [173] and present a significantly increased risk of developing type 2 diabetes [174]. Hyperinsulinaemia derived from insulin-resistant states would further stimulate the intrinsically increased androgen biosynthesis, characterising theca cells of PCOS ovaries [175]. In turn, androgen excess, observed in 60 to 80% of women suffering from PCOS [176] and further exacerbated by abdominal obesity, might play a crucial role in preferentially determining the expansion of visceral adipose tissue, contributing to IR and thus creating a vicious cycle ([172].

#### 7.1.1. Chemerin

Chemerin has been discovered as a novel adipokine associated with obesity and metabolic syndrome in 2007 [64]. Its strong interaction with insulin metabolism is evident. Indeed, insulin profoundly enhances chemerin secretion from adipose tissue [177], while chemerin has been demonstrated to modulate insulin signalling and glucose disposal in in vitro and animal studies [178], inducing an insulin-resistant state in both adipocytes and skeletal muscle [142].

**Figure 11:**
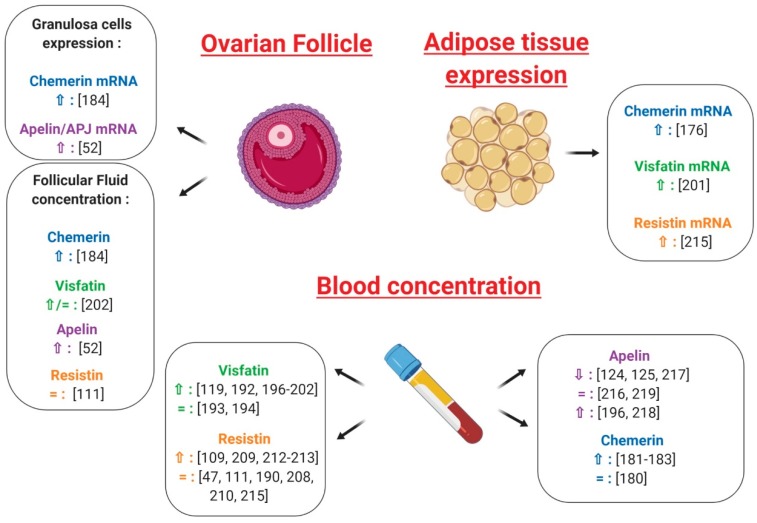
Effects of chemerin, visfatin, resistin and apelin on ovarian physiology, plasma and adipose tissue in polycystic ovarian syndrome (PCOS) as compared to control patients. ⇧ Increase/stimulation. ⇩ Decrease/inhibition.

Independently from insulin and obesity, the existence of a correlation between Chemerin and PCOS has repeatedly been evoked [179]. Serum and ovarian levels of chemerin have been shown to be elevated in a dihydrotestosterone (DHT)-induced rat PCOS model [99] and, despite some discordant data [180], most of the authors report higher plasma chemerin concentrations in PCOS women [177,181,182,183] (Figure 11). These patients also seem to present increased chemerin expression in the subcutaneous and omental adipose tissue [177] (Figure 11); very recently, Wang et al. demonstrated that chemerin follicular fluid concentration and its mRNA levels in granulosa cells were higher in PCOS normal-weight patients than in controls [184] (Figure 11), a finding that we have confirmed subsequently in our laboratory (Bongrani et al., in press).

**Figure 12:**
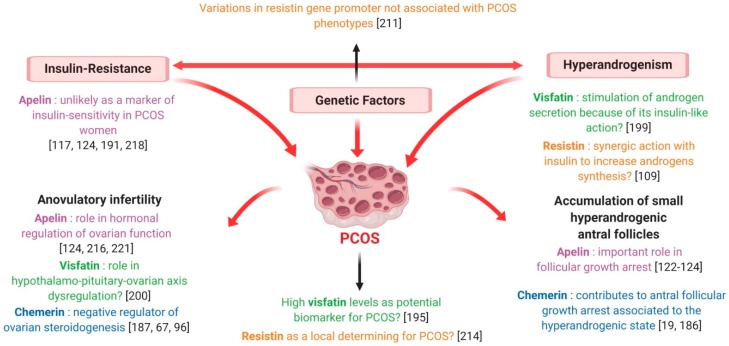
Description of PCOS syndrome and possible involvement of chemerin, visfatin, resistin and apelin in this syndrome.

Interestingly, circulating chemerin levels were greater in PCOS hyperandrogenic women compared to the euandrogenic ones [180] and positively correlated with free androgen index [180] and total testosterone levels [184]. Furthermore, in DHT-treated rats, CMKLR1 gene deletion protected against the negative effects of chronic androgen treatment on progesterone secretion, cycling and ovulation [185]. As testosterone treatment has been demonstrated to up-regulate the expression of chemerin and its receptors in vitro [184], Lima et al. suggested that negative androgen effects on ovary could be partly mediated by this adipokine [186] (Figure 12). Indeed, hyperandrogenism would increase ovarian chemerin, which, in turn, functions as a chemoattractant ligand for blood monocytes expressing CMKLR1. Inflammatory monocyte-derived CMKLR1+ M1 macrophages, attracted to chemerin-rich ovarian follicles, would induce granulosa cell apoptosis contributing to the antral follicular growth arrest associated with the hyperandrogenic pro-inflammatory state characteristic of PCOS [186] (Figure 12). It is noteworthy that elevated chemerin levels in the DHT-induced rat PCOS model were positively related to increased granulosa cell apoptosis [19] and that women presenting higher chemerin concentrations in follicular fluid had significantly fewer oocytes and lower high-quality embryo rates [184], suggesting that chemerin could actually be involved in folliculogenesis disruption at the origin of PCOS [19] (Figure 12).

As described in paragraph 5.1, the expression of chemerin and its receptors in human ovarian cells has been largely demonstrated [67] and the role of the chemerin/CMKLR1 pathway in follicle function and steroidogenesis has repeatedly been evoked [185]. Indeed, chemerin acts as an important negative regulator of ovarian steroidogenesis, inhibiting the IGF-1-induced secretion of progesterone and oestradiol in human granulosa cells [67] and suppressing the FSH-induced expression of aromatase and P450scc in cultured rat pre-antral follicles and granulosa cells [99,187]. Remarkably, chemerin concentration has been shown to be higher in follicular fluid than in plasma [67] and, in contrast to serum chemerin, the rise of follicular chemerin in women with PCOS has been demonstrated to be independent of changes in adiposity [186]. Thus, chemerin regulation at the ovarian level seems to be different from the systemic one, suggesting that this adipokine could play a paracrine and/or autocrine regulatory role in the ovary. Moreover, by negatively affecting steroidogenesis and participating in androgen induction of antral follicle growth arrest, chemerin seems to be strongly involved in the pathogenesis of anovulatory infertility characterising PCOS patients [186] (Figure 12).

#### 7.1.2. Visfatin

Despite some conflicting results [188,189,190], a recent meta-analysis revealed that plasma visfatin levels are significantly increased in subjects presenting overweight/obesity, IR, metabolic syndrome and cardiovascular diseases [191], with visfatin expression seeming to be modulated by some insulin-sensitising agents. Indeed, 3 month-treatment with metformin in PCOS women resulted in a significant decrease in serum visfatin concentration [192]. Although two studies failed to highlight a significant difference between PCOS and healthy women [193,194] (Figure 11), most of the authors, and particularly the recent Sun et al. meta-analysis [195], found significantly higher plasma visfatin levels in PCOS patients [123,192,196,197,198,199,200,201,202] (Figure 11). Similarly, in PCOS women, visfatin expression in adipose tissue was increased independent of BMI [201] (Figure 11) and visfatin concentrations in follicular fluid were similar [123] or higher [202] in comparison with BMI-matched normally ovulatory women (Figure 11). Interestingly, the adipose tissue of PCOS women is characterised by an up-regulating alteration of lipolysis due to a selective increase in the function of protein-kinase A hormone-sensitive lipase complex [203] and 50% higher responsiveness to norepinephrine then healthy adipose tissue [203]. Since visfatin seems to be released during the lysis of fat cells rather than being secreted [204], the increased visfatin concentrations observed in PCOS women are possibly derived from this up-regulated lipolysis [123]. Visfatin is also known to display pro-inflammatory properties and immune functions [78,205] and, within adipose tissue, it has been demonstrated to be secreted not only by adipocytes, but also by inflammatory cells, such as macrophages [199]. As PCOS is characterised by low-grade inflammation, this could additionally explain its association with higher visfatin levels.

Interestingly, Panidis et al. found a positive correlation between circulating visfatin and plasma LH, evoking a possible role of this adipokine in the hypothalamo-pituitary-ovarian axis dysregulation observed in PCOS [200] (Figure 12). It is also noteworthy that serum visfatin levels have been found to be significantly higher in PCOS hyperandrogenic women than in the euandrogenic ones [193] and in PCOS hirsute adolescents when compared with non-hirsute patients [194]. Furthermore, despite some discordant results [123,195], a positive association between circulating visfatin and markers of hyperandrogenism has repeatedly been highlighted [193,194,198,200], even independent from insulin-sensitivity and other confounding factors [199]. As previously described, visfatin exerts an insulin-mimetic action and insulin can stimulate theca cell androgen synthesis, notably in IR-related hyperinsulinaemic conditions [193]. Thus, visfatin could be implicated in PCOS pathogenesis by influencing ovarian androgen secretion because of its insulin-like action [199] (Figure 12).

In conclusion, whether visfatin plays a role in the physiopathology of PCOS remains a matter of debate and further studies are needed to elucidate its significance. However, high visfatin levels seem to be an intrinsic characteristic of PCOS, suggesting that this adipokine could be a potential biomarker for this syndrome [195].

#### 7.1.3. Resistin

Interestingly, in a randomised placebo-controlled study involving overweight women suffering from PCOS, circulating levels of resistin were significantly decreased by the insulin-sensitising agent rosiglitazone [206]. However, although a significant correlation between plasma resistin and IR or type 2 diabetes has been reported [207], most of the studies do not confirm the existence of such an association [47,115,208,209,210] (Figure 11). In particular, Panidis et al. showed that plasma resistin levels did not differ between PCOS and control normal-weight women, even though the former were more insulin-resistant. Furthermore, after stepwise multiple regression analysis, circulating resistin was not associated with any parameter independent of BMI, suggesting that it correlated with IR as a consequence of obesity itself rather than as an independent causative factor [47] (Figure 11).

As discussed above, resistin involvement in human female reproduction is known. However, at present, the existence of an association between resistin and PCOS is largely debated. Indeed, variations in the resistin gene promoter were not associated with PCOS phenotypes [211] (Figure 12) and data concerning plasma resistin levels in PCOS women are inconsistent. While some authors pointed out significantly higher resistin concentrations in the plasma of PCOS patients [112,209,212,213] (Figure 11), no difference between PCOS and healthy women has been reported by several others [47,115,190,208,210,214] (Figure 11). Similarly, resistin concentrations in follicular fluid did not differ between normal-weight PCOS and healthy women [115] (Figure 11). On the contrary, resistin mRNA levels in adipocytes have been found to be twice as high in PCOS patients compared to controls [214] (Figure 11) and significantly decreased after laparoscopic ovarian electrocautery [215], suggesting that, although systemic resistin does not seem to be actively involved in PCOS pathogenesis, it may act as a local determining factor for this syndrome [47,214] (Figure 12).

Remarkably, in humans, Seow et al. found greater mRNA resistin levels in the adipocytes of PCOS women presenting higher serum testosterone levels [214] (Figure 11) and the study of Munir et al. showed that circulating resistin in the PCOS group, but not in controls, was positively correlated with plasma testosterone [112]. Thus, it has been suggested that some important differences in polycystic ovaries may facilitate the responsiveness of theca cells to resistin, which may synergise with insulin to increase androgen synthesis [112].

In conclusion, in light of the data collected so far, resistin does not seem to be a major determining factor in PCOS pathogenesis. However, its role in ovarian theca cells and, notably, in androgen production deserves to be further investigated.

#### 7.1.4. Apelin

Data about the existence of an association between circulating apelin and IR in PCOS are still inconsistent. Indeed, plasma apelin levels have been found to correlate negatively [216], but also positively [196] with HOMA-IR (Homeostatic Model Assessment for Insulin Resistance), and most studies have excluded the existence of any significant association between this adipokine and IR [128,191,217,218]. It is therefore unlikely that apelin could represent a marker of insulin-sensitivity in women with PCOS.

In our laboratory, we repeatedly found that apelin concentration in follicular fluid and levels of apelin and its receptor APJ mRNA in granulosa cells are higher in PCOS patients than in healthy controls [52] (Figure 11). More conflicting data are, however, available for circulating apelin in PCOS women, with an almost equal number of authors reporting significantly lower [128,129,217], higher [196,218] or unchanged [216,219] levels compared to control women (Figure 11).

Interestingly, in our laboratory we recently demonstrated that, independent of PCOS diagnosis, apelin and APJ expression in granulosa cells and follicular fluid is increased in women presenting a high number of ovarian small antral follicles resulting from the failure in selection of a dominant follicle (Bongrani et al., in press). Hence, according to these data, apelin could be significantly involved in PCOS pathogenesis, markedly contributing to the arrest of follicular development (Figure 12). It is currently admitted that folliculogenesis disruption observed in PCOS is derived from an increased responsiveness of small follicles to FSH in terms of oestradiol and progesterone synthesis, inducing a premature responsiveness to LH [220]. As a consequence, PCOS anovulatory women present higher LH and oestradiol levels, as well as lower FSH concentrations, than those in the normal early follicular phase [220]. Interestingly, in rats, apelin is expressed in the arcuate supraoptic and paraventricular hypothalamic nuclei and suppresses LH, FSH and prolactin secretion [221]. Furthermore, in women with PCOS a negative correlation between plasma apelin and LH levels has repeatedly been found [128,216] (Figure 12), strongly suggesting a role of this adipokine in the hormonal regulation of ovarian function, especially with regard to follicular development.

In conclusion, current knowledge strongly supports the involvement of apelin in follicular growth arrest and hypothalamus-pituitary-ovary axis perturbations at the origin of ovulatory dysfunction typically associated with PCOS, encouraging further studies about the role of this adipokine in reproductive function.

### 7.2. Gestational Diseases

During pregnancy, the placenta secretes cytokines, including TNF-alpha, IL-6 and IL-1β, and increases both their local and systemic levels, which is believed to be important in determining foetal allograft fate [222]. As described in paragraph 6.3, several adipokines, such as adiponectin, leptin, resistin, visfatin and apelin, are also secreted by the placenta [223] and have been implicated in metabolic adaptations to normal gestation, as well as in preeclampsia and other complications of pregnancy [224]. Moreover, some of these molecules are postulated to play a significant role in creating a favourable environment for implantation and placental development [222]. In contrast to adiponectin and leptin, studies about the regulation of chemerin, visfatin, resistin and apelin in gestational diabetes, preeclampsia and intrauterine growth restriction are limited. Furthermore, data are mostly descriptive, not allowing clarification of the physiological significance of the dysregulation of these novel adipokines in pregnancy complications.

#### 7.2.1. Gestational Diabetes Mellitus

Gestational diabetes mellitus (GDM) is defined as a carbohydrate intolerance first detected in pregnancy, which affects approximately 14% of pregnancies worldwide (International Diabetes Federation. IDF Diabetes Atlas, 8th ed. Brussels, Belgium, 2017). It poses serious risks for the mother and developing foetus. Moreover, although it usually resolves following delivery, in the long-term, women with a past history of GDM and babies born of GDM pregnancies are at an increased risk of obesity, type 2 diabetes mellitus and cardiovascular diseases [225]. Pregnancy is a unique condition characterised by transient physiological IR, which progresses with advancing gestation, aimed at facilitating delivery of nutrients to the foetus. In fact, slightly elevated glycaemia makes glucose available to be transported across the placenta to fuel foetal growth. IR also promotes endogenous hepatic glucose production and lipolysis in adipose tissue, resulting in a further increase in blood glucose and free fatty acid concentrations [225]. Pregnant women compensate for these changes through hypertrophy and hyperplasia of pancreatic β-cells, as well as increased glucose-stimulated insulin secretion [226]. Failure of this compensatory response gives raise to maternal hyperglycaemia or GDM [227]. Thus, GDM is usually the result of β-cell dysfunction on a background of chronic IR during pregnancy. IR is mainly attributed to placental hormones and increased maternal adiposity, although the underlying mechanisms are not fully understood [223]. Recently, several new potential mediators of IR have been identified and, among these, adipokines seem to play a key role [223].

##### Chemerin

**Figure 13:**
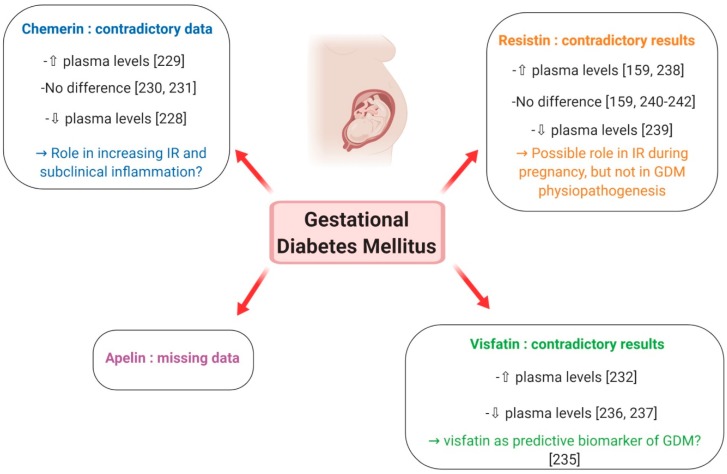
Putative involvement of plasma levels of chemerin, visfatin, resistin and apelin in gestational diabetes mellitus. ⇧ Increase/stimulation. ⇩ Decrease/inhibition.

Chemerin has been suggested to constitute an insulin-sensitising factor which may counteract IR [228]. Contrary to normal pregnancy, which is associated with increased plasma chemerin levels that decrease post-partum, Hare et al. showed that circulating chemerin was reduced in GDM women and did not change with the normalisation of glucose tolerance following delivery [228] (Figure 13). However, a recent meta-analysis by Zhou et al., evaluating 11 studies carried out between 2010 and 2017 and including a total of 742 GDM patients and 840 normal pregnant women [229], reported that the overall levels of plasma chemerin in GDM women were significantly increased when compared with healthy pregnant ones and that this difference was more evident in the second- than in the third-trimester. The authors thus concluded that chemerin may play a powerful role in the pathophysiology of GDM by increasing IR and promoting subclinical inflammation [229] (Figure 13). These results are, however, contradicted by a previous review, evaluating various adipokines in GDM, which demonstrated that adiponectin, leptin and TNF-alpha are more likely than chemerin, resistin and visfatin to play a role in the pathogenic mechanism of GDM [230] (Figure 13). Finally, no difference in chemerin levels, either in the early third trimester or at delivery, was reported between healthy and GDM women by Van Poppel et al. [231] (Figure 13). They instead demonstrated that, compared to infants from healthy mothers, newborns from GDM patients had higher chemerin levels in arterial but not venous cord blood, probably reflecting a higher pro-inflammatory status in the foetus of pregnancies complicated by GDM [231]. In summary, although chemerin seems to contribute to the increasing IR and subclinical inflammation that is characteristic of GDM, at present, data concerning its role in the physiopathology of this pregnancy complication are still controversial and do not allow a unique conclusion.

##### Visfatin

Visfatin promotes adipogenesis and exerts insulino-mimetic effects [223]. In normal pregnancy, it may improve insulin sensitivity during the second and third trimesters and its up-regulation in the IR state during gestation may be part of a physiological feedback mechanism to improve insulin signalling [223]. However, there are huge discrepancies in reports about visfatin levels in pregnancies complicated by GDM. Higher serum visfatin levels in GDM women compared to normal glucose tolerance controls were observed in a nested case-control study, but no relationship with fasting plasma glucose, insulin, IR or BMI could be obtained [232] (Figure 13). On the contrary, two cross-sectional studies found that, in women with GDM, circulating visfatin was positively correlated with fasting and post-glucose load insulin in the third trimester [233], and that GDM was independently associated with increased maternal plasma visfatin concentrations [234]. Subsequently, Ferreira et al. reported increased levels of visfatin in the first trimester of women who later developed GDM, suggesting that this adipokine could be a biomarker of this pregnancy complication [235] (Figure 13). However, many other studies reported lower visfatin levels in GDM patients [236,237] (Figure 13). Differences in study design, including the number of patients, gestational age and BMI, may contribute to explain the different results between studies. In conclusion, further research is still needed to clarify the relationship between visfatin and obesity and any causal association with IR and GDM. In particular, prospective studies are required to evaluate whether visfatin can be predictive of GDM.

##### Resistin

The role of resistin in managing glucose homeostasis remains unclear, as most studies have failed to show a correlation between plasma resistin levels and insulin sensitivity in humans [223]. Resistin is expressed in the human placenta and, in normal pregnancy, it is up-regulated in the third trimester, possibly contributing to the decrease in insulin sensitivity [160]. In GDM, the studies available so far did not give unique results. Circulating resistin has been shown to be elevated in women with GDM compared to normal pregnancies [161,238] (Figure 13). Nevertheless, Kuzmicki et al. could not demonstrate an independent relationship between serum resistin concentration and insulin levels or IR [238]. Contrary to these results, another study found lower resistin levels in GDM patients in comparison with euglycaemic controls [239] (Figure 13) and most case-control studies reported no difference in circulating resistin levels in women with and without GDM [162,240] (Figure 13), a finding further confirmed in a large prospective study [241] (Figure 13) and in a recent meta-analysis [242] (Figure 13). Moreover, no difference was reported in resistin release from placental and subcutaneous adipose tissue obtained from normal pregnant subjects and GDM women [243]. Collectively, current data suggest that resistin may mediate IR during pregnancy, but it is unlikely to have a central role in glucose homeostasis and the development of GDM.

##### Apelin

Apelin is known to be an adipokine implicated in glucose homeostasis, and is found to be increased in obese and T2DM individuals [240]. Although its presence has been documented in human placental tissue [164], very few studies have addressed the expression of apelin in human pregnancy. An increase in apelin fat mRNA expression was observed only in early gestation, suggesting that apelin is not associated with hyperinsulinaemia of late pregnancy, but rather related to adipose tissue accumulation [223]. In GDM, cross-sectional studies of circulating apelin have contradictory results, including unchanged and increased levels [230] (Figure 13). Moreover, no association between plasma apelin concentration or apelin/APJ mRNA expression and GDM or the indices of IR was noted in a study involving 101 GDM patients and 101 women with normal glucose tolerance between 24 and 32 weeks of gestation and 20 GDM and 16 healthy controls at term [244]. In light of these data, it seems thus unlikely that apelin is directly involved in GDM physiopathogenesis.

#### 7.2.2. Preeclampsia

Preeclampsia (PE) is a pregnancy complication affecting 4.6% of pregnant women worldwide [245] and a major contributor to foetal, neonatal and maternal morbidity and mortality [246]. It may develop from 20 weeks of gestation up to 6 weeks postpartum [246] and is characterised by the de novo development of arterial hypertension associated with one of the following features: proteinuria, maternal organ impairment (comprising renal insufficiency, hepatic cytolysis, neurological complication or haematological disorder like thrombocytopenia or haemolysis) and uteroplacental dysfunction, including foetal growth restriction [247]. Indeed, PE is associated with small-for gestational age (SGA) neonates in 10–25% of cases [248]. The pathophysiology of PE has not yet been fully elucidated, but abnormal placentation and imbalance between angiogenic and anti-angiogenic factors (including VEGF, PIGF (Placental growth factor), soluble fms-like tyrosine kinase 1 and endoglin) appear to be major contributors [222]. Initial incomplete trophoblast invasion and abnormal uterine spiral artery remodelling induce uteroplacental ischemia. This is followed by the release of placental factors, such as inflammatory cytokines and reactive oxygen species, into maternal circulation; these are able to trigger a broad intravascular inflammatory response, which is another essential step for the development of PE [247]. Like GDM, PE shares risk factors with metabolic syndrome including IR, subclinical inflammation and obesity [248] and women with a history of hypertensive pregnancy disorders present a 1.4 to 3-times higher risk of future cardiovascular diseases compared to women with normotensive pregnancies [249].

##### Chemerin

**Figure 14:**
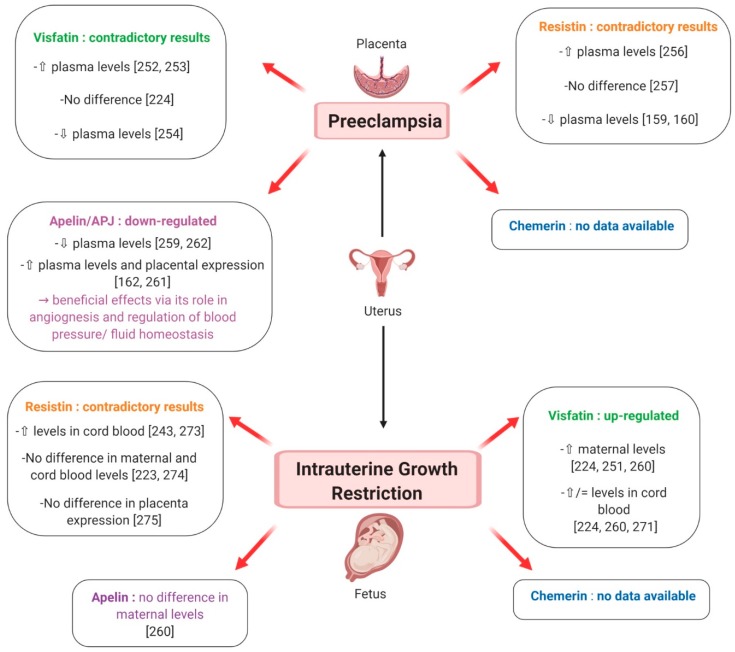
Putative involvement of plasma levels of chemerin, visfatin, resistin and apelin in preeclampsia and intrauterine growth restriction. ⇧ Increase/stimulation. ⇩ Decrease/inhibition.

Despite its high expression in human placenta [74] and its well-known association with obesity, glucose metabolism and metabolic syndrome [64], data concerning the possible involvement of chemerin in physiological and pathological pregnancy is really poor. To the best of our knowledge, only one study in a small number of subjects has investigated chemerin in PE. Interestingly, maternal serum chemerin concentrations were significantly increased in preeclamptic women compared to healthy pregnant ones [250] (Figure 14). Furthermore, patients with severe PE had higher chemerin levels than those with mild PE, and circulating chemerin was independently correlated with markers of dyslipidaemia and PE severity, suggesting that chemerin regulation of glycolipid metabolism may contribute to the pathogenesis of this pathological condition [250] (Figure 14). Chemerin is also known as a pro-inflammatory adipokine, induced by TNF-alpha and enhancing macrophage adhesion [19], and as a potent angiogenic factor, inducing gelatinolytic activity of endothelial cells [64,154]. Hence, its role in PE might be more relevant and involve other aspects of its physiopathology, which deserve more in depth investigation.

##### Visfatin

The presence of visfatin transcript and protein has been detected in human foetal membranes and placenta [154] and pregnant women present higher circulating visfatin levels compared to non-pregnant subjects [234]. Notably, in normal pregnancies, median maternal plasma visfatin concentrations peak in the second trimester of gestation, between 19 and 26 weeks, and show a nadir between 27 and 34 weeks [234,251]. In women with PE, circulating visfatin levels have been reported to be increased [252,253] (Figure 14), similar [224] (Figure 14) or decreased [254] (Figure 14) compared to healthy pregnant women. Notably, Fasshauer et al. found that, after multivariate analysis, PE remained a significant predictor of circulating visfatin, independent of HOMA-IR and BMI [252]. Moreover, in the Mazaki-Tovi et al. study of patients with PE, there was no significant difference in maternal visfatin concentration between those with or without an SGA neonate, suggesting that the effect of FGR, which is known to increase visfatin levels (as described later), was overwhelmed by PE [224]. The authors proposed that in the presence of common risk factors for PE and SGA, high maternal visfatin concentrations could have beneficial metabolic effects, resulting in increased insulin-sensitivity and thus protecting mothers from PE [224]. Nevertheless, Hu et al. reported contrasting results, i.e. markedly decreased visfatin levels in preeclamptic women, irrespective of their BMI, and speculated an involvement of this adipokine in the exaggerated IR that characterises this pathological condition [254] (Figure 14). In light of these data and because visfatin is known to improve glucose tolerance, it has been suggested that the up-regulation of maternal visfatin concentration in IR-associated pregnancy complications may be part of a physiological feedback mechanism aimed at improving insulin signalling [223,251]. Furthermore, visfatin expression has recently been shown to be significantly related to TNF-alpha and IL-6 mRNA expression in placental tissues [223] and recombinant visfatin treatment of human foetal membranes causes a significant increase in inflammatory cytokines including IL-1β, TNF-alpha and IL-6 [154]. Thus, elevated visfatin levels observed in PE could additionally be associated with the pro-inflammatory state characteristic of this and other pregnancy complications.

##### Resistin

Resistin is expressed in human placenta, and mainly in trophoblastic cells [246]. Plasma resistin levels in pregnant women are significantly higher compared to those in non-pregnant controls and resistin gene expression, as well as its circulating concentrations, is more prominent as pregnancy advances [160]. As resistin gene expression is higher in the placenta than in adipose tissue, where it remains unchanged throughout pregnancy [160], it has been postulated that placental resistin may contribute to the physiological decrease in insulin-sensitivity occurring in the second half of human pregnancy [255]. Data about resistin levels in PE are discordant. Indeed, Haugen et al. reported higher resistin concentrations in women affected by PE compared to normal pregnant controls [256] Figure 14). However, such a difference was lost after controlling for IR and, as resistin placental gene expression was unchanged, it probably mostly depended on altered renal function in preeclamptic patients, which contributes to elevate resistin plasma concentrations [256]. In contrast to this report, Cortelazzi et al. and Chen et al. found that circulating resistin levels were lower in preeclamptic women than in the normotensive healthy pregnant ones, possibly due to reduced placental production because of the smaller size of the placenta [161,162] (Figure 14). Finally, Hendler et al. failed to find a difference in serum resistin concentrations between pregnant women with and without PE [257] (Figure 14). Hence, according to these data, resistin does not seem to be the main determinant of IR observed in PE [223]. However, as resistin is known to exert pro-inflammatory effects and stimulates IL-6 and TNF-alpha synthesis [258], it may be involved in the exaggerated maternal inflammatory response associated with PE pathogenesis.

##### Apelin

Because of its cardiovascular protective action and its role in the regulation of fluid homeostasis and insulin metabolism, the apelin/APJ system has been supposed to play a role in adaptation to pregnancy and regulation of foetal growth [259]. Additionally, embryonic expression studies in animals indicate that apelin is an important angiogenic factor required for normal blood vessel growth and endothelial cell proliferation [223]. Nevertheless, reports studying the expression and the function of this adipokine in human pregnancy are very few. Although apelin is known to be up-regulated in obesity and hyperinsulinaemia states in humans and mice [50], apelin fat mRNA expression seems to increase only in the early period in pregnant rats, suggesting that it may not be associated with physiological IR occurring in late pregnancy [223]. Furthermore, a recent study analysing maternal apelin concentrations in pregnant women failed to show any significant correlation between apelin and insulin levels [260]. In light of these data, apelin contribution to the regulation of maternal insulin sensitivity therefore seems limited. Nevertheless, several recent works have highlighted the important role of apelin in PE physiopathology. Indeed, although some authors showed higher maternal levels [261] (Figure 14) and increased placenta expression [164] (Figure 14) of apelin in preeclamptic pregnant women compared to healthy ones, most of the reports demonstrated a down-regulation of the apelin/APJ system in hypertensive pregnant disorders [259,262] (Figure 14). Notably, Yamaleyeva et al. found 30% lower content of apelin in the chorionic villi of preeclamptic patients compared to villi obtained from women with a normal gestation and proposed that lower apelin levels in the preeclamptic placenta might diminish its opposing modulation of vasoconstrictor mediators, which results in the increased blood pressure defining PE [259]. Indeed, the blood pressure-lowering action of apelin is well documented in several animal models and in patients with heart failure [263] and apelin is known to act on vascular smooth muscle cells, inducing either vasodilatation or vasoconstriction via different pathways [259]. Additionally, in the study of Yamaleyeva et al., apelin release from the chorionic villi was reduced by angiotensin II administration, suggesting a potential interaction between apelin and the renin-angiotensin system [259]. The beneficial effects of apelin in PE have also been suggested by a very recent report using a rat model in which PE symptoms were induced by reducing uterine perfusion pressure. In this model, apelin treatment significantly reversed the elevation in blood pressure and increased the total foetal weight, resulting in higher embryo survival rates [263]. Interestingly, these effects were obtained by improving the impaired eNOS/NO (endothelial nitric oxide synthase/nitric oxide) signalling pathway and preventing the activation of oxidative stress, which is one of the key features of PE pathogenesis [263].

Noteworthy, very recently, another component of the apelin-APJ system, named elabela has been postulated to be actively involved in PE development. Indeed, elabela is a new endogenous peptide ligand for the APJ receptor, which seems to play a crucial role in embryonic development [229]. Ho et al. demonstrated that deletion of the *elabela* gene in mice caused PE-like symptoms and that the administration of elabela to pregnant *elabela*-null mice prevented the increase in maternal blood pressure, proteinuria and FGR [264]. The authors thus proposed that the loss of *Elabela* might perturb early placental development, resulting in its inadequate perfusion. However, whether this effect is obtained via a paracrine role in angiogenesis or, indirectly, by inducing vasodilatation and regulating fluid balance, is currently unknown [264,265]. It needs to be underlined that these results have not been confirmed in humans, as no difference was found in placental transcript abundance or circulating levels of elabela in preeclamptic women compared to controls [266].

In conclusion, because of its involvement in angiogenesis and the regulation of blood pressure and fluid balance, the apelin/APJ system might play an important role in PE pathogenesis, encouraging further studies about its physiological relevance in pregnancy and its complications.

#### 7.2.3. Intra-Uterine Growth Retardation

Foetal growth restriction (FGR) is defined by an estimated foetal weight less than the 10th percentile for the population at a given gestational age. It is a common complication of gestation, affecting up to 25% of pregnancies in low- to middle-income countries [267]. It derives from a placental failure to adequately supply oxygen and nutrients to the developing foetus, thus resulting in stunted foetal growth [268]. This phenomenon, named placental insufficiency, is idiopathic in up to 60% of cases and it is due to a physiological deficiency in uterine spiral arteries remodelling, resulting in restricted uteroplacental perfusion. In the foetus, hypoxia results in so-called brain-sparing, which is the preferential blood flow redistribution to vital organs like the brain, myocardium and adrenal glands, inducing a decrease in foetal weight and an altered foetal organ development that are associated with increased rates of neonatal mortality and morbidity [268]. Interestingly, in addition to placental insufficiency, FGR shares with PE several mechanisms of disease, including the anti-angiogenic state, the increased maternal intravascular inflammatory response [224] and the excessive maternal IR [269]. Furthermore, SGA neonates are more likely to develop metabolic complications, such as glucose metabolism disorders and adipose tissue dysfunction, later in life [270]. Several maternal adipokines are already known to link maternal nutrient status and adipose tissue metabolism to placental nutrient transport, thus contributing to foetal organ development and growth patterns in utero [222].

##### Chemerin

Although chemerin is actively involved in glucose and lipid metabolism [64] and its expression is elevated in the human placenta [74], its role in foetal growth has poorly been studied and, to the best of our knowledge, no report about chemerin involvement in FGR is currently available.

##### Visfatin

Given its insulin-mimetic action and as its expression in human placenta is limited to the villous capillary of the foetal endothelium, visfatin has been proposed to play a role in the transfer of glucose from the maternal to the foetal circulation [246]. The existence of a relationship between this adipokine and foetal growth has further been suggested by recent findings of elevated visfatin concentrations in cord blood [255]. Remarkably, all of the authors studying visfatin in the third trimester of pregnancies complicated by FGR reported higher maternal levels compared to control pregnant women with appropriate-for-gestational-age infants [224,251,260] (Figure 14). Visfatin has thus been proposed as a novel marker that is up-regulated in pregnant women with SGA neonates [251] (Figure 14). With regard to visfatin concentration in cord blood, however, data are more controversial. Indeed, while Ibanez et al. and Malamitsi-Puchner et al. found higher visfatin levels in SGA neonates compared to the appropriate-for-gestational-age ones [260,271] (Figure 14), Mazaki-Tovi et al. failed to highlight any difference between these two groups [224] (Figure 14). Similarly, the latter authors reported that visfatin concentration in foetal circulation was lower than in the maternal one and observed no significant correlation between the two [224] (Figure 14). This finding is in contrast with Malamitsi-Puchner et al., who showed that maternal and foetal visfatin concentrations were similar and significantly correlated each other [260] (Figure 14). Interestingly, Mazaki-Tovi et al. proposed that increased visfatin levels in SGA neonates might be linked to their different and/or altered visceral adiposity, which seems to contribute to the development of IR and impaired glucose metabolism in adulthood [224,272] (Figure 14).

##### Resistin

As resistin gene expression is higher in term placenta than in first trimester placental tissue and its levels in cord blood samples are elevated, resistin has been postulated to be involved in the control of foetal energy expenditure and deposition of adipose tissue [255]. Indeed, resistin is supposed to act as a feedback regulator of adipogenesis, exerting an inhibitory effect on adipose conversion [255]. Cord serum resistin levels have been found to be higher in growth-restricted pregnancies and lower in macrosomic foetuses when compared to normal ones [243,273] (Figure 14), further suggesting the existence of a negative correlation between maternal and cord blood resistin and birth weight. However, these results are not confirmed by other authors, who demonstrated no difference in resistin maternal [223] (Figure 14) and cord blood [274] (Figure 14) concentrations, as well as in placental resistin expression [275] (Figure 14) between FGR and normal pregnancies. Furthermore, in the study of Yeung et al., who analysed adipokines in newborn dried blood spots, resistin was inconsistently associated with birth size after accounting for the other measures [276]. In light of the currently available data, therefore, it is difficult to speculate on a specific role for resistin in foetal energy metabolism.

##### Apelin

Apelin and APJ have been identified at high levels in human placenta and, notably, in cytotrophoblasts, syncytiotrophoblasts and foetal endothelial cells, suggesting that apelin may have a paracrine action on human chorionic villi [164]. As apelin concentration is twice as high in umbilical cord blood than in maternal circulation [260] (Figure 14) and a positive correlation between maternal and foetal plasma apelin was observed in full-term normal pregnancies, a transplacental transfer of this adipokine with a potential impact on foetal growth has been suggested [259]. Notably, Mayeur et al. demonstrated that maternal intravenous apelin administration increased transplacental transport of glucose in a rodent model, either inducing the vasodilatation of placental vessels or, indirectly, modulating maternal blood pressure [167]. Interestingly, reducing the food intake of rat mothers significantly decreased their apelin levels, while a higher apelin expression was observed at the foeto-maternal interface, evoking a possible compensatory response of FGR foetuses to increase their glucose supply and improve their growth [167]. In light of these data, apelin seems to therefore have a beneficial role in foetal glucose homeostasis. However, despite such promising results in animals, to the best of our knowledge, only one study in humans has investigated this adipokine in pregnancies complicated by FGR and it did not find any difference in maternal apelin levels between FGR and normal pregnancies [260].

## 8. Adipokines and Male Reproductive Pathologies

In recent years, interest in the role of novel adipokines in male fertility has steadily grown. Indeed, a recent meta-analysis suggested that resistin and visfatin affect spermatogenesis [277] and a new study demonstrated that long treatment of human cultured Sertoli cells with chemerin, visfatin and resistin at high concentrations, which are often observed in obese men, significantly suppressed FSH receptor expression and up-regulated that of the cytochrome P450 CYP26A1, inducing a phenotype characteristic of the pre-pubertal state [278]. Thus, it has been postulated that these adipokines negatively affect Sertoli cell maturation, possibly contributing to testis dysfunction and fertility perturbations associated with obesity [278]. However, although the presence of chemerin and visfatin has been detected in the human testis, data concerning the pathogenic role of novel adipokines in male reproductive disorders are currently very poor.

With regard to chemerin, Thomas et al. reported that its concentration in seminal fluid was negatively correlated with spermatic motility and positively correlated with sperm concentration [139], while Bobjer et al. showed a negative correlation between plasma chemerin and LH, oestradiol and SHBG levels [279]. Furthermore, they found that, even after adjusting for BMI, serum chemerin concentration was lower in subfertile men compared to healthy controls, suggesting that, despite its positive association with BMI, this adipokine is independently linked to reproductive function [279]. Interestingly, vasectomised patients presented lower sperm chemerin levels than healthy men [139], evoking a local secretion of chemerin in the male genital tract, although chemerin levels in seminal fluid have repeatedly been found to be significantly lower than in blood plasma [135,139].

In light of the current knowledge, the expression of resistin in the testis has been detected only in rodents [20]. However, collectively, the studies carried out in men suggest a negative role of this adipokine in male fertility. Indeed, while some authors found no significant correlation between plasma resistin levels and sperm parameters [139], our laboratory recently showed that circulating resistin is negatively correlated with sperm vitality and normomorphic sperm percentage [135]. This finding agrees with the results obtained by Moretti et al., who highlighted a negative correlation between resistin levels in seminal fluid and sperm vitality and motility [280]. Very interestingly, they also demonstrated that seminal resistin was increased in patients presenting a leukocytospermia or a varicocele, two pathological conditions characterised by a local pro-inflammatory state, and positively correlated with levels of pro-inflammatory markers such as elastase, TNF-alpha and IL-6 [280]. Resistin could thus represent a marker of inflammation in the male genital tract and its increased levels in pathological situations like leukocytospermia might be related to alterations in sperm parameters [20].

Although visfatin has been demonstrated to be produced by human spermatozoa, notably the immature ones [137], and its levels are 100 times higher in seminal fluid than in blood plasma [139], no consistent data are available regarding its role in male reproductive disorders. In a very recent study carried out in obese and diabetic rats, plasma visfatin was negatively correlated with semen quality parameters, testosterone and LH levels and degenerative changes in the testis, suggesting that this adipokine may play a role in the physiopathology of male infertility associated with obesity and diabetes [281]. It must, however, be kept in mind that several authors failed to find any significant correlation between seminal and plasma visfatin and sperm parameters in humans [139].

As for visfatin, the literature for apelin is very poor and limited to animal studies. Notably, Sandal et al. demonstrated that the intracerebroventricular infusion of apelin-13 in male rats significantly suppressed LH release and decreased the number of Leydig cells, both resulting in a reduction in plasma testosterone levels [143]. However, at present, this possible role of apelin in the central regulation of male reproduction has not been confirmed, especially in humans.

## 9. Conclusions

Adipokines (chemerin, resistin, visfatin and apelin) and their cognate receptors (CMKLR1, GPR1, CCRL2 for chemerin and APJ for apelin) are expressed in peripheral tissues but also in the reproductive tract (from hypothalamus-pituitary, gonads) in both males and females of different species, including humans. Thus, these hormones could contribute to regulate the reproductive functions and consequently participate to explain some reproductive disorders. However, until now, most of the data come from in vitro experiments, and in vivo studies are limited. Sometimes, the in vitro data are contradictory, which can be explained by differences depending on the species but also by the use of different concentrations of tested adipokines and/or different experimental design. Transgenic mice where these adipokines or adipokine receptors are specifically deleted in the reproductive cells do not yet exist. Therefore, it is difficult to discriminate the effects of local adipokines production from systemic production. Moreover, most of these adipokines exist under various forms (ex-chemerin and apelin) and until now it has not been possible to detect all of these specific forms. Thus, the concentration in plasma as well as in the follicular fluid and seminal plasma of these various forms of adipokines as well as their effect in the reproductive tract remain to be investigated. For chemerin, some recombinant forms and specific enzyme-linked immunosorbent assays (ELISAs) have been developed in humans and mice but future studies are needed to further define the potential mechanistic role of these isoforms in reproduction. Interestingly, plasma and/or tissue expression of chemerin, resistin, visfatin and apelin might be associated with various female reproductive disorders including PCOS syndrome, gestational diabetes, preeclampsia, and uterine growth restriction. The involvement of these adipokines has been less studied in male reproductive pathologies. Finally, all of the data suggest that additional studies are necessary to better understand the role and molecular mechanism of adipokines in the control of fertility in order to potentially use them as prognostic markers and/or therapeutic targets in different reproductive disorders.

## Abbreviations

ACE2	Angiotensin converting enzyme 2
ADSF	Adipocyte-specific secretory factor
Akt	Retroviral *oncogene also named Protein Kinase B*
AMPK	AMP-activated Protein Kinase
APJ	Tissue Inhibitors of MetalloProteinases
APLNR	Apelin receptor
BAT	Brown Adipose Tissue
BeWo	Placental cell line
BMI	Body Mass Index
CCRL2	C-C Motif Chemokine Receptor Like 2
ChemR23	Chemerin Receptor 23
CHO cells	Chinese hamster ovary cells
CL	Corpus Luteum
CMKLR1	Chemokine-Like Receptor 1
COV 434	Cellosaurus cell line 43 (immortalized ovarian cells)
CSF	CerebroSpinal Fluid
DCN	Decorin
DHT	Dihydrotestosterone
E2	Estradiol
ERK1/2	Extracellular signal-Regulated Kinases 1 & 2
EVTs	Extravillous *trophoblasts*
FSH	Follicle Stimulating Hormone
Gata4	GATA binding protein 4.
GnRH	Gonadotropin-Releasing Hormone
GPCR	G protein-coupled receptors
GPR1	G protein-coupled receptor 1
hCG	human Chorionic Gonadotropin
HCR	C-terminal receptor binding domain
HIV	Human Immunodeficiency Virus
HSD3B	3β-Hydroxysteroid dehydrogenase
HUVECs	Human umbilical vein endothelial cells
IGF-1	Insulin like Growth Factor 1
IGF-1-R	Insulin-like growth factor 1 receptor
IL17/22	Interleukin 17/22
Insl3	Insulin-like 3
IR	Insulin Resistance
KGN	Steroidogenic human granulosa-like tumor *cell* line
LH	Luteinizing Hormone
MAPK	Mitogen-Activated Protein Kinases
mTOR	mammalian Target Of Rapamycin Complex 1 or mechanistic target of rapamycin
NAD	Nicotinamide Adenine Dinucleotide
NadV	Nicotinamide phosphoribosyltransferase NadV
NAMPT	Nicotinamide phosphoribosyltransferase
NFkB	Transcription factor Nuclear Factor-kappa B
NK cells	Natural Killer cells
NMN	Nicotinamide MonoNucleotide
ORF	Open reading frame
P4	Progesterone
P450 CYP26A1	Cytochrome P450 CYP26A1
PBEF	Pre-B-cell colony-enhancing factor
PBMCS	Peripheral Blood Mononuclear Cells
PE	PreEclempsia
PGF2alpha	Prostaglandin F2alpha
PIGF	Placental growth factor
PKA	Protein Kinase A
PKC	Protein Kinase C
PPAR	Peroxisome Proliferator-Activated Receptor
PPAR gamma	Peroxisome proliferator-activated receptor gamma
PTEN	Phosphatase and TENsin homolog
PTX sensitive	Pertussis Toxin sensitive
*RARRES2*	Retinoic Acid Receptor Responder protein 2
Retn	Resistin
RhoA	Ras homolog gene family, member A
ROR1	Receptor tyrosine kinase-like orphan receptor 1
SF1	Steroidogenic Factor 1
SHBG	Sex Hormone-Binding Globulin
SIRT1	Member of the sirtuin family
SIV	Simian Immunodeficiency Virus
StAR	Steroid Acute Regulatory protein
STAT3	Signal transducer and activator of transcription 3
T2DM	Type 2 Diabetes Mellitus
TLR4	Toll Like Receptor 4
TNF alpha	Tumor Necrosis Factor alpha
VEGF	Vascular endothelial growth factor
VEGFR2	Vascular endothelial growth factor receptor 2
WAT	White Adipose Tissue
WT	Wild Type
